# Pre-clinical characterisation of E2814, a high-affinity antibody targeting the microtubule-binding repeat domain of tau for passive immunotherapy in Alzheimer’s disease

**DOI:** 10.1186/s40478-020-0884-2

**Published:** 2020-02-04

**Authors:** Malcolm Roberts, Ioanna Sevastou, Yoichi Imaizumi, Kavita Mistry, Sonia Talma, Madhurima Dey, Jane Gartlon, Hiroshi Ochiai, Zhi Zhou, Shigeru Akasofu, Naoki Tokuhara, Makoto Ogo, Muneo Aoyama, Hirofumi Aoyagi, Kate Strand, Ezat Sajedi, Kishan Lal Agarwala, Jared Spidel, Earl Albone, Kanta Horie, James M. Staddon, Rohan de Silva

**Affiliations:** 1 Hatfield Research Laboratories, Eisai Limited, Hatfield, UK; 2 Reta Lila Weston Institute & Department of Clinical and Movement Neurosciences, UCL Queen Square Institute of Neurology, 1 Wakefield Street, London, UK; 3 Tsukuba Research Laboratories, Eisai Co., Tsukuba-shi, Ibaraki, Japan; 4 Eisai Inc., Welsh Pool Road, Exton, PA USA

**Keywords:** Tau, Alzheimer, tauopathy, immunotherapy, neurodegeneration

## Abstract

Tau deposition in the brain is a pathological hallmark of many neurodegenerative disorders, including Alzheimer’s disease (AD). During the course of these tauopathies, tau spreads throughout the brain via synaptically-connected pathways. Such propagation of pathology is thought to be mediated by tau species (“seeds”) containing the microtubule binding region (MTBR) composed of either three repeat (3R) or four repeat (4R) isoforms. The tau MTBR also forms the core of the neuropathological filaments identified in AD brain and other tauopathies. Multiple approaches are being taken to limit tau pathology, including immunotherapy with anti-tau antibodies. Given its key structural role within fibrils, specifically targetting the MTBR with a therapeutic antibody to inhibit tau seeding and aggregation may be a promising strategy to provide disease-modifying treatment for AD and other tauopathies. Therefore, a monoclonal antibody generating campaign was initiated with focus on the MTBR. Herein we describe the pre-clinical generation and characterisation of E2814, a humanised, high affinity, IgG_1_ antibody recognising the tau MTBR. E2814 and its murine precursor, 7G6, as revealed by epitope mapping, are antibodies bi-epitopic for 4R and mono-epitopic for 3R tau isoforms because they bind to sequence motif HVPGG. Functionally, both antibodies inhibited tau aggregation in vitro*.* They also immunodepleted a variety of MTBR-containing tau protein species. In an in vivo model of tau seeding and transmission, attenuation of deposition of sarkosyl-insoluble tau in brain could also be observed in response to antibody treatment. In AD brain, E2814 bound different types of tau filaments as shown by immunogold labelling and recognised pathological tau structures by immunohistochemical staining. Tau fragments containing HVPGG epitopes were also found to be elevated in AD brain compared to PSP or control. Taken together, the data reported here have led to E2814 being proposed for clinical development.

## Introduction

Tauopathies are a group of diverse, age-associated neurodegenerative disorders that are neuropathologically characterised by various neuronal and glial inclusions of abnormally hyperphosphorylated, insoluble and fibrillar tau protein [31]. These diseases include Alzheimer’s disease (AD), the most common neurological disorder, as well as the parkinsonian primary tauopathies, progressive supranuclear palsy (PSP) and corticobasal degeneration (CBD). Currently, only symptomatic treatments are available for AD, giving rise to a huge unmet clinical need for new therapies to slow down or, better, halt progression of the disease.

AD is pathologically defined by extracellular amyloid plaques consisting of Aβ peptide fragments, derived from the β-amyloid precursor protein (APP), and intraneuronal tau inclusions in the form of neurofibrillary tangles (NFTs) and neuropil threads. Extensive autopsy studies [61] have contributed to the current hypothesis that amyloidosis precedes and accelerates neocortical tau pathology, which together contribute to cognitive decline in AD [39]. Furthermore, Aβ pathology generally occurs years, if not decades, prior to onset of clinical symptoms, posing considerations for therapeutic intervention. Early work by Braak and colleagues showed tau pathology burden and distribution better reflected disease severity, progression and cognitive decline compared with the initiating amyloid load [8]. In more recent years, it has emerged that the different tauopathies are initiated by distinct pathological molecular triggers allowing release of abnormal tau conformers (“seeds”) as agents of intercellular propagation of tau pathology. Following tau deposition, neuronal malfunction and death ensue, leading to clinical progression of disease [20].

**Fig. 1:**
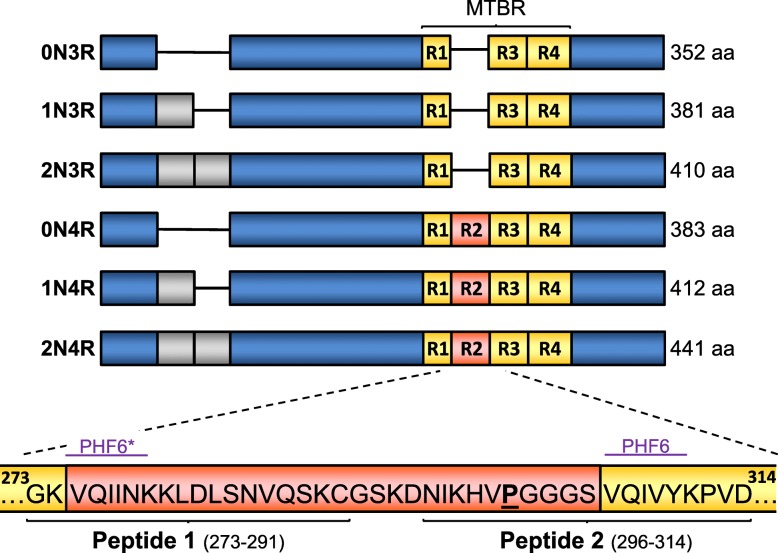
Peptide selection for antibody generation. A schematic representation of all six tau isoforms expressed in human adult brain is shown. The two hexapeptide sequences necessary to initiate tau aggregation, PHF6* and PHF6, are also indicated. To generate potential therapeutic antibodies, two peptide immunogens contained within the microtubule-binding region (MTBR) of 4R tau isoforms were selected. Peptide 1 (273–291) contained two amino acids within the R1 region with the remainder in R2 encompassing the PHF6* motif. Peptide 2 (296–314) contained ten amino acids within R2 and nine in R3, also including the PHF6 motif as well as the P301 residue (bold and underlined), often introduced as mutated in pre-clinical research models. Amino-acid numbering according to largest (2N4R) isoform (NP_005901)

Tau is a multifunctional protein with a primary role of mediating assembly and stability of axonal microtubules (MTs) [13]. Six isoforms of tau protein are expressed in healthy adult human brain as a result of alternative splicing. These isoforms contain 0, 1, or 2 N-terminal inserts and, through splicing of exon 10, 3 (3R-tau) or 4 (4R-tau) imperfect repeat sequences [34, 40, 41, 50, 75] which constitute the microtubule-binding region (MTBR) in the carboxy-half of the protein (Fig. 1). Two related hexapeptide motifs, PHF6* (_275_VQIINK_280_; numbering according to 2N4R-tau isoform; Accession Code: NP_005901.2) and PHF6 (_306_VQIVYK_311_), located within the second-(R2) and third (R3) MT-binding repeat domains respectively (Fig. 1), are necessary for the pathological aggregation of tau [47, 77, 78]. These short motifs are hydrophobic with a high propensity for β-sheet structure formation [68, 70]. The different 3R- and 4R-tau isoforms are thought to play an important role in pathology, with the composition of tau aggregates varying between different tauopathies. AD has approximately equal amounts of 3R- and 4R-tau contained within pathological structures whereas other tauopathies, such as corticobasal degeneration (CBD) or Pick’s disease exhibit, respectively, predominantly 4R- or 3R-tau pathology (for review, see [18]).

During the course of disease, tau becomes hyperphosphorylated and released from MTs, resulting in a conformational conversion of tau from its highly soluble, natively unfolded state to the insoluble fibrillar aggregates by way of oligomeric intermediates [29]. Pathological conformers of tau (the “seeds”) can, on contact, facilitate the templated conversion of normal soluble tau into pathological conformers, leading to a cascade-like amplification and spread of pathology [49]. Functional tau seeds require an intact MTBR [22]. This region also forms the protease-resistant core of the terminal filamentous deposits [26], highlighting the importance of the MTBR in disease. Furthermore, many cases of familial frontotemporal dementia (FTD) have causative missense mutations in the tau gene (MAPT) that result in coding changes in amino acids predominantly clustered around the tau MTBR, reducing MT-binding capacity and leading to a greater propensity of tau to aggregate [78]. Transmissible seeds have also been reported to spread transsynaptically to neighbouring neurons via interconnected pathways, leading to the hierarchical propagation of tau pathology in disease-specific patterns [9, 69]. Effective ways of slowing or halting tau disease progression may therefore be through prevention of either pathological conversion of tau into functional seeds or blocking inter-cellular spread of tau protein conformers that contain the MTBR.

The precise molecular nature of the MTBR-containing seed that transmits from cell to cell is unknown. It has been suggested that seeds are oligomers or short fibrils as opposed to higher-order assemblies [44]. However, recent work has shown monomeric seed-competent tau could be isolated from AD brain [57]. This seed-competent tau differs in intramolecular interactions when compared to inert, soluble monomeric tau; it is predicted that such interactions could expose the two PHF6 motifs that are otherwise buried in the soluble tau [57]. Conversely, the PHF6 motifs in inert tau are predicted to be shielded by a preferential hairpin conformation around this region making them less prone to aggregation [57].

The possibility that toxic tau seeding species containing the MTBR are extracellular and transmissible in human brain opens up the prospect of therapeutic intervention through immunotherapy. Since the first preclinical report of active immunisation in transgenic mice [5], this area of tau research has received increasing attention. Several passive and active immunotherapies targeting either different regions of the tau protein, pathological conformation, phospho-epitopes, oligomeric or aggregated tau have been described, with some now in early-stage clinical trials (for reviews see [14, 45]). Since the MTBR of tau plays such an important role in forming functional seeds as well as being structurally key and integral to the core of the end stage fibrils, we decided to generate therapeutic antibodies against that region. In order to prevent seeding and the adoption of filament-forming structures within tau, we therefore sought to identify antibodies that could precisely target and mask the MTBR PHF6 motifs and/or flanking inter-repeat regions necessary for conformational conversions and aggregation. The goal was to identify antibodies that may prevent tau becoming seed-competent. Such agents could slow the initiation or progression of pathological tau formation rather than specifically recognise later-stage pathological conformations. E2814 is described here as a humanised, bi-epitopic, high affinity antibody specifically binding the HVPGG sequences within R2 and R4 of the tau MTBR. R2 is present only in 4R-tau isoforms, directly adjacent to the PHF6 domain in R3. The creation and preclinical development of E2814 based on its murine precursor, 7G6 is described in this report. Clinical trials to evaluate the safety, tolerability and efficacy of E2814 in human subjects have been initiated.

## Materials and methods

### Nomenclature

Tau isoforms with three or four microtubule-binding repeats are designated: 3R-tau and 4R-tau, respectively. The repeats are labelled, starting from the N-terminal side: R1, R2, R3 and R4 (Fig. 1).

### Animals

Animal care and experimental procedures were performed in an animal facility accredited by the Health Science Center for Accreditation of Laboratory Animal Care and Use of the Japan Health Sciences Foundation. All protocols were approved by the Institutional Animal Care and Use Committee and carried out in accordance with, as appropriate, the Animal Experimentation Regulations of Eisai Co., Ltd or Cell Engineering Corporation.

### Postmortem brain

*Post-mortem* fixed and frozen brain samples were obtained from the Queen Square Brain Bank for Neurological Disorders, (UCL Queen Square Institute of Neurology, London). Ethical approval for the study was obtained from the Local Research Ethics Committee of the National Hospital for Neurology and Neurosurgery, London, UK. Tissue was stored for research purposes under license 12,198 from the Human Tissue Authority, UK.

### Antibody generation and purification

All original mouse hybridomas were generated by Cell Engineering Corporation (Osaka, Japan). Based on tau sequence, Peptide 1 (_273_GKVQIINKKLDLSNVQSKC_291_; numbering according to 2N4R (441 amino-acids)) and Peptide 2 (_296_NIKHVPGGGSVQIVYKPVD_314_) (Fig. 1) were selected, synthesised (with an N-terminal cysteine added to Peptide 2 for coupling) and coupled to keyhole limpet hemocyanin (KLH) using m-maleimidobenzoyl-N-hydroxysuccinimide ester (MBS) chemistry. Peptide-conjugated KLH was then mixed with Freund’s complete adjuvant (1:2 v/v), injected into the tailbase of *Mapt*-null mice (Jackson Labs, 007251) and, 3 weeks later, lymphocytes were isolated from the medial iliac lymph nodes and fused with mouse myeloma SP2 cells. Resulting hybridomas were maintained in growth media: Hybridoma-SFM (Gibco), supplemented with 10% foetal bovine serum (FBS, Biosera), 1 ng/mL human IL-6 (R&D Systems) and 1x penicillin-streptomycin-amphotericin B suspension (Wako). Culture supernatants were screened for antibodies using standard or competitive ELISA formats with BSA-conjugated peptide or recombinant wild type 2N4R tau protein (Enzo Life Sciences). Cultures were serially-diluted to obtain single-cell clones for expansion and cryopreservation. From multiple clones, four of interest were selected that were raised against Peptide 1 and eight against Peptide 2. The 7G6 clone derived from Peptide 2 immunisation was sequenced and selected for further characterisation and humanisation based on its high affinity for recombinant tau protein.

Heavy and light chain human sequences derived from the original 7G6 hybridoma were subcloned into proprietary (Eisai Inc.) mammalian expression vectors and transiently expressed by co-transfection into HEK293 cells using an Expifectamine™ 293 transfection kit (Thermo Fisher Scientific). All details of antibody humanization are described in patent WO2019077500 wherein E2814 is designated 7G6-HCzu25-LCzu18. Antibodies were purified by Protein-A affinity chromatography, eluted in 100 mM glycine-HCl (pH 2.9) and desalted into formulation buffer (25 mM sodium phosphate pH 6.5, 150 mM NaCl).

### Recombinant tau protein production

All recombinant truncated and full-length tau proteins (other than those used in Additional file 1: Figure S1) were produced in *E.coli* and purified as described previously [72] with minor modification. Briefly, cDNA encoding tau sequences were subcloned into the pET15b vector (Novagen) and transformed into BL21 (DE3) cells (ThermoFisher). Following induction of protein expression, cell pellets were resuspended in 50 mM PIPES pH 6.4, 1 mM EGTA, 1 mM DTT and protease inhibitors. Cells were disrupted through sonication and the clarified lysate was then boiled for 15 min to allow enrichment of soluble and un-precipitated tau protein in the supernatant. Further purification was performed by ammonium sulfate precipitation, Cellufine™ phosphate (JNC Corporation) ion exchange chromatography and reverse-phase HPLC.

### Antibody affinity determination

Binding affinities of wild-type recombinant 2N4R tau protein for murine 7G6 and human E2814 antibodies were determined by surface plasmon resonance (SPR) using a streptavidin capture method and Biacore T^− 100^ instrument (GE Healthcare). Each antibody was buffer-exchanged into 0.1 M sodium bicarbonate pH 8.3 and then biotinylated by incubation with freshly-prepared NHS-PEG4-biotin at a 5:1 M ratio (biotin:antibody) for 1 h at room temperature. Excess biotin was removed by two sequential buffer exchanges into PBS using 0.5 mL Zeba SPIN 40 kDa MWCO desalting columns (Thermo Fisher Scientific). Prior to use in the SPR assay, biotinylated antibodies were diluted to 2 μg/mL in PBS containing 0.2% BSA. Antibodies were injected at a flow rate of 10 μL/min to achieve capture levels of approximately 225 response units (RU) on a prepared CAP chip (with final streptavidin levels of 3500 RU) from a biotin CAPture kit (GE healthcare). Recombinant wild-type 2N4R tau protein was passed over the antibody-bound chips at concentrations of 0, 0.16, 0.8, 4 and 20 nM. Once kinetic data had been collected, a 1:1 Langmuir model was employed to calculate the association rate, dissociation rate and equilibrium dissociation constants for each biotinylated antibody.

Similarly, the binding affinity and mode of interaction kinetics of the unmodified murine 7G6 antibody for recombinant human 2N4R wild-type and mutant P301S tau were also assessed by SPR but on a Biacore S200 (Molecular Interaction Technology). A protein A/G chip was used to capture the 7G6 antibody. Sensorgrams were then obtained for the association and dissociation of the varying concentrations of recombinant human 2N4R tau ranging from 1.25 to 20 nM for the wild-type and 2.5 to 40.0 nM for the P301S mutant protein. Responses were expressed as RU to represent molecular mass changes on 7G6 captured by the protein A/G during the reaction time course (association for 120 s; dissociation for 380 s). The association rate, dissociation rate and equilibrium dissociation rate were calculated as described above.

### Fine epitope mapping

Purified murine 7G6 and human E2814 antibodies were epitope-mapped to full length human wild-type tau protein (NCBI reference P10636–8) by PEPperPRINT (Heidelberg, Germany). The tau protein sequence was elongated in silico with a neutral GSGSGSG linker sequence at both the N and C-termini. Overlapping 15-mer peptides covering the entire in silico elongated sequence were synthesised and then printed in duplicate onto a glass chip. This chip also contained 82 additional spots of an HA-tag control peptide (YPYDVPDYAG). 7G6 and E2814 anti-tau antibodies were diluted to 1 μg/mL in PBS containing 0.05% Tween-20 and 10% Rockland blocking buffer (Rockland, MB-070). Each antibody was then incubated separately as described above for 16 h at 4 °C with shaking. Primary antibodies were removed and each chip was washed in PBS containing 0.05% Tween 20. Wash buffer was removed and, as appropriate, secondary antibodies (LI-COR) were added: either (for 7G6) goat anti-mouse IgG (H + L) DyLight™680 (1:5000), or (for E2814) goat anti-human IgG (H + L) DyLight™680 (1:5000) together with (for the control peptide) anti-HA tag IgG DyLight™ 800 (1:2000). All secondary antibodies were diluted in the same buffer as primary antibodies and then incubated on chips for 45 min at room temperature. The secondary detection antibodies were removed and the chips were washed again. Fluorescence images were acquired on the LI-COR Odyssey™ Imaging System. Microarray data were then analysed using the PepSlide™ Analyser software.

### In vitro tau aggregation

Prior to inducing tau aggregation, wild-type or P301S recombinant 2N4R tau protein was reduced to prevent effects on aggregation through disulphide bond formation [6]. To achieve and maintain thiol reduction, a buffer containing 60 μM recombinant tau, 0.5 mM TCEP, 25 mM HEPES (pH 7.4) and 100 mM NaCl in a final volume of 20 μL was prepared, heated to 98 °C for 30 min and cooled to room temperature.

The next step was addition of relevant antibodies or buffer controls. First, the reduced tau protein was diluted by addition of 52 μL buffer containing 25 mM HEPES (pH 7.4) and 100 mM NaCl. Then, 1 μL of 100x HALT® protease inhibitor cocktail (Thermo Fisher Scientific) and 25 μL of 5 mg/mL antibody working stock solution (or in some cases 25 μL of buffer) were added to the mixture. The mixture was then incubated at 37 °C for 30 min. Tau aggregation was initiated by addition of 2 μL of a 3 mg/mL heparin stock (MW ≈ 5000; Thermo Fisher Scientific). The final reaction components in a total volume of 100 μL were: 12 μM tau, 0.1 mM TCEP, 1.25 mg/mL (8.3 μM) antibody, 25 mM HEPES (pH 7.4), 100 mM NaCl, 1x HALT® Protease Inhibitor cocktail and 0.06 mg/mL (≈ 12 μM) heparin. Reactions were maintained at 37 °C over a period of 6 days after heparin addition.

At required time points, 10 μL aliquots of the tau aggregation assay mixture were removed and placed directly into a well of a 384-well plate (781,076, Greiner). Then, 20 μL of a 15 μM thioflavin S (Sigma) working solution was added to give a final concentration of 10 μM in the well. The plate was incubated in the dark for 30 min at room temperature. Green fluorescence (485 nm excitation and 520 nm emission), indicative of tau aggregation, was measured on a Pherastar® plate reader (BMG Labtech).

### Cell-based tau seeding assay

Truncated tau (244–372,‘K18’) [60] fibrils were generated by mixing recombinant monomeric protein with 2 mM DTT and 240 μg/mL heparin (Acros) in 100 mM sodium acetate, pH 7.0 at 37 °C for 48 to 96 h. Aggregates were collected by ultracentrifugation and then resuspended in 100 mM sodium acetate, pH 7.0. The resulting fibrils were sonicated prior to use as seeds. Recombinant 2N4R P301S tau monomer was also used as seed material in separate experiments.

To prepare the immunodepletion samples, the E2814 antibody was used to precipitate fibrillar K18 or monomeric recombinant P301S tau protein using the Immunoprecipitation Dynabeads® Protein G kit (Thermo Fisher Scientific) as per the manufacturer’s instructions. Briefly, different amounts of E2814, human IgG1 (BioXCell, BP0297) control antibody or buffer (25 mM sodium phosphate pH 6.5, 150 mM NaCl) were pre-incubated with 1.5 mg of Dynabeads. Antibody-bound (or buffer-treated) beads were resuspended in buffer with or without 0.2% BSA. Either 300 ng of K18 fibrils or 30 ng of P301S recombinant tau were added to the bead suspension before mixing head-over-end for 30 min at room temperature. Following separation of the magnetic beads, unbound material was collected and retained as the immunodepleted sample for use on cells.

Lenti-X 293 T cells were transiently transfected with the pcDNA3.1(+) vector encoding the human mutant P301S 0N4R tau isoform, using LTX and Plus™ Reagent (Life Technologies) as per manufacturer’s recommendation. Cell suspension was dispensed into polyethylenimine-coated 96 well plates (0.8 to 1.2 × 10^3^ cells/well) in Dulbecco’s Modified Eagle’s Medium (D-MEM) containing 10% heat-inactivated FBS. Cells were incubated overnight at 37 °C under a 5% CO_2_ atmosphere. The following day, each sample was diluted into Opti-MEM (Life Technologies). Plated cells were washed twice and left in half the original volume of Opti-MEM. An equal volume of diluted immunodepleted sample was added to each well and incubated for 48 h at 37 °C under a 5% CO_2_ atmosphere. Each experiment was performed in triplicate or quadruplicate. Following a 2 day incubation period, cells were fixed in 4% paraformaldehyde and stained with Cellstain® DAPI solution (1:1000, Dojindo; DAPI diluted in 5% BSA in TBS) and Thioflavin S (ThS, Thioflavin S dissolved in 50% Ethanol to a final concentration of 0.0003%). Each well was then washed twice with 50% ethanol, and then again with purified water prior to imaging the plate. Fluorescent images of each well were obtained on the Opera Phenix High Content Screening System (Perkin Elmer). Numbers of ThS and DAPI-positive signals in each well were analyzed using Harmony High-Content Analysis Software (Perkin Elmer). The seeding effects of samples added to cells were calculated using Microsoft Excel, TIBCO Spotfire software and GraphPad Prism.

### Intrahippocampal tau seed injection in mice

Aggregated recombinant human 2N4R P301S tau seeds were prepared in the same way as described for K18 fibrils used in the cell-based assay. Either 3 μL of P301S tau seeds (at 1.5 mg/mL) or 100 mM sodium acetate pH 7.0 (buffer control) was stereotactically injected into the left hippocampus of 3 to 4 month old male *MAPT* P301S transgenic mice [3]. The rate of seed injection was 0.5 μL/min using an UltraMicroPump III and Micro4 Controller (World Precision Instruments). More than 6 h prior to intrahippocampal seed administration, mice were injected intraperitoneally with either 7G6 or IgG_2b_ control (BioXCell, BP0086) antibodies at 40 mg/kg or vehicle (25 mM sodium phosphate pH 6.5, 150 mM NaCl). Peripheral dosing at the same levels was performed 1 and 2 weeks following the intrahippocampal seed addition.

One week after the final peripheral antibody or vehicle administration, the animals were sacrificed. Terminal blood was collected for plasma analysis, as well as CSF, before each animal was perfused with saline. Brain tissue was removed and the cortex and hippocampus from each side (ipsilateral and contralateral sides to the seed injection site) were isolated, weighed and immediately frozen in liquid nitrogen before storage at − 80 °C.

To quantify the extent of tau seeding and transmission, levels of sarkosyl-insoluble tau were measured. Sarkosyl (N-lauroylsarcosinate) has been used in tau research for many years to enrich for end-stage fibrils found in human tauopathy brains as well as transgenic mice [33, 35]. The majority of tau protein in brain is soluble but tau fibrils are insoluble in sarkosyl detergent. Here, dissected brain tissues were homogenized on ice in 19 volumes (w/v) of extraction buffer containing 50 mM Tris-HCl (pH 7.5), 5 mM EDTA, 1 mM EGTA, 1% NP-40, 0.25% deoxycholic acid sodium salt, 0.1 M NaCl, 0.5 mM PMSF, 1x PhosSTOP (Roche) and 1x Complete (EDTA(−)) protease inhibitor cocktail (also Roche). Homogenates were centrifuged at 163,000 x *g* at 4 °C for 20 min. The pellet was then resuspended in 10 volumes (original tissue weight/volume) of buffer containing 10 mM Tris-HCl (pH 7.5), 0.5 M NaCl, 1 mM EGTA, 10% sucrose and 1% sarkosyl prior to sonication. Sarkosyl-treated samples were incubated at 37 °C for 60 min and then centrifuged at 163,000 x *g* at 4 °C for a further 20 min. The final pellet was resuspended in 10x volumes (original tissue weight/volume) of PBS to constitute the sarkosyl-insoluble fraction.

The amount of tau in the sarkosyl-insoluble fraction was quantified by western blotting. Sarkosyl-insoluble fractions were solubilized in NuPAGE LDS sample buffer (Invitrogen) containing reducing agent and then heated at 80 °C for 10 min. Proteins were resolved on 12.5% polyacrylamide gels and then transferred to 0.2 μm PVDF membranes (Bio-Rad). Blots were blocked in 2.5% skimmed milk in TBS containing 0.05% Tween for 1 h at room temperature and then probed with the human-specific monoclonal anti-tau antibody HT7 (1:1000, ThermoFisher Scientific) also for 1 h at room temperature. Blots were washed and then incubated with an HRP-conjugated, anti-mouse secondary antibody (1:2000, GE Healthcare) for a further hour at room temperature. Tau proteins were detected by addition of chemiluminescent substrate (Merck Millipore) and images captured using the Fusion FX system (Vilber-Lourmat). Bands were quantified by reference to a serial dilution of tau-containing samples on each blot derived from the sarkosyl-insoluble fraction of spinal cord from 8 month old animals of the same transgenic mouse strain. An arbitrary unit (AU) of 1 was defined as the band intensity detected by the HT7 antibody from 7 μg of the spinal cord insoluble fraction loaded on the gel.

### Immunohistochemistry with E2814

Immunohistochemistry was performed on fixed, paraffin-embedded sections (8 μm) from de-identified Alzheimer’s disease (AD; frontal cortex; *n* = 1), progressive supranuclear palsy (PSP; frontal cortex; *n* = 1) and Pick’s disease (PiD; hippocampus; *n* = 1) cases. The sections were dewaxed using several changes of xylene, followed by washes in several changes of 100% industrial methylated spirit (IMS) for several minutes each change. Sections were then incubated with hydrogen peroxide (H_2_0_2_) in methanol (2 ml H_2_0_2_ per 100 ml methanol) for 10 min at room temperature to block endogenous peroxidase and then rinsed under running tap water for 10 min. For antigen retrieval, sections were pressure-cooked in citrate buffer pH 6 for 10 min, rinsed under running tap water followed by a rinse in Tris-buffered saline (TBS) solution. To block non-specific human-on-human antibody reactivity, staining was performed using the Klear Human HRP-Polymer DAB Detection Kit (D103–18, GBI Labs) as per supplier’s protocol with some modifications. On the day before use, E2814 antibody or control human IgG_1_ antibody (Clone X9G6C80, Eisai Inc.) was diluted in Human Primer (RTU) Reagent at 0.5 μg/mL, 0.33 μg/mL and 0.2 μg/mL and then mixed gently for 30 s to 1 min and stored at 4 °C overnight. The pre-incubated antibody in Human Primer (RTU) Reagent/antibody mix was raised to room temperature and quenched with 1x Reagent 2 (Quenching Buffer 5x) for 15 to 30 min at room temperature before placing on ice for no more than 1 h. Prior to primary antibody incubation, sections were blocked for non-specific binding following the supplier’s protocol. The antibody in Human Primer (RTU) Reagent/Quenching Buffer was then added to cover each section and incubated for 60 min at room temperature, followed by three washes in PBS/0.05% Tween-20. Sections were then incubated with 1:1000 dilution of HRP-conjugated anti-human antibody (sc-2907, SantaCruz) either for 30 min at room temperature or overnight at 4 °C. For HRP staining, avidin biotin complex (ABC, Dako) was prepared following the manufacturer’s instructions. Sections were rinsed three times for 5 min each in TBS before applying the ABC working solution to all sections for 30 min at room temperature. The ABC solution was removed and tissue was washed again in TBS as before. A DAB working solution (1 ml of 5% DAB in 100 ml TBS) was prepared and activated by adding H_2_0_2_ (32 μL per 100 mL DAB working solution). Sections were immersed in activated DAB solution for 5 min at room temperature. Finally, the tissue was rinsed in TBS and then extensively under running tap water for 10 min. The sections were counterstained in Mayer’s Haematoxylin for 30 s to 1 min and then washed again under warm running tap water for approximately 10 min. Sections were dehydrated through ascending grades of IMS (70, 90 and 100%), cleared in xylene and mounted under DPX medium (Sigma-Aldrich). Microscopy and digital image acquisition of stained sections were carried out on a Nikon Eclipse Ni Microscope and images processed using Adobe Photoshop®.

### Sarkosyl-extraction of tau fibrils from brain samples

Frozen brain tissue was homogenized with a hand-held TissueRuptor® (Qiagen) in 10x v/w ice-cold buffer containing 10 mM Tris-HCl pH 7.5, 0.8 M NaCl and 10% sucrose, supplemented with protease and phosphatase inhibitors (cOmplete™ and PhosSTOP™, Roche). Homogenates were centrifuged at 16,000 x *g* at 4 °C for 20 min, supernatants were collected and the pellets re-homogenized in 5x v/w of ice-cold buffer and centrifuged again under the same conditions. The two supernatants were combined and adjusted to 1% N-lauroylsarcosinate (sarkosyl) (w/v) (Sigma) and then agitated at room temperature for 1 h. Each sample was then ultracentrifuged at 100,000 x *g* at 4 °C for 1 h. Resulting sarkosyl-insoluble pellets were resuspended in ice-cold PBS (0.6 mL per g of starting material) and stored at 4 °C until use.

### Immunogold staining of brain sarkosyl-insoluble fraction tau

Sarkosyl-insoluble fraction samples containing the tau fibrils were placed onto the center of a formvar/carbon-coated 400 mesh nickel grid for 2 min. The grids were then incubated for 5 min on a 25 μL drop of blocking buffer: 1% horse serum, 1% BSA, 0.1% Tween-20 and 0.1% sodium azide in PBS. For pronase-treated samples, prior to the initial blocking, grids were incubated on a drop of 0.4 mg/mL of pronase (Sigma-Aldrich), for 4 min, followed by a brief wash in blocking buffer. Blocking was followed by incubation on a 25 μL drop of primary antibody, diluted in blocking buffer, overnight at 4 °C. The control Tau-5 antibody [64] (Thermo Sciences) used for double-labelling is a pan-tau antibody recognising amino acids 218–225 within the mid-domain of 2N4R tau. Following primary antibody incubation, grids were washed on a series of 3 drops of blocking buffer for 10 min on each drop. Grids were then incubated for 1.5 h on a 25 μL drop of secondary antibody conjugated to either 6 nm or 12 nm colloidal gold particles diluted in PBS. Following incubation, the grids were washed on 2 drops of PBS for 30 s each, followed by a 5 min incubation on 2% glutaraldehyde in PBS to cross-link antibodies to other proteins. Fixation was followed by two 30 s washes in PBS and finally each sample was negatively stained by incubation on a drop of 2% phosphotungstic acid in 20 mM phosphate buffer for 30 s exactly. Samples were left to dry and then viewed under a Jeol 1010 transmission electron microscope with Digital Image Capture.

### LC/MS analysis of tau peptides in human tauopathy brain

Frozen brain tissue from AD cases (*n* = 3; Braak stage VI, frontal cortex), progressive supranuclear palsy cases (*n* = 4; PSP, temporal cortex) and control brains (*n* = 2; frontal cortex) were crushed in liquid nitrogen, weighed and then homogenised in 10 volumes of PBS containing HALT protease and phosphatase inhibitors (Thermo Fisher Scientific) with 20 strokes in a Dounce homogeniser on ice. Samples were centrifuged at 5250 x *g* for 30 min at 4 °C. Half the supernatant was retained. The remaining supernatant and pellet were re-homogenised and centrifuged as before. The two supernatants were pooled to create the clarified ‘whole homogenate’ for each sample. To obtain the insoluble fraction, whole homogenate samples were ultracentrifuged at 100,000 x *g* at 4 °C for 1 h. The insoluble pellet was resuspended in 0.2 mL of lysis buffer: 7 M Urea, 2 M thio-urea, 3% CHAPS, 1.5% n-octyl glucoside, 100 mM triethyl ammonium bicarbonate (TEAB) and 50 mM DTT. The suspension was sonicated and then, with protection from light, alkylated with 0.1 mL of 500 mM iodoacetamide for 30 min at room temperature. For protein digestion, the Filter-Aided Sample Preparation (FASP) method [80] was employed with minor modifications. Briefly, protein solutions were loaded onto a Nanosep 10 K filter unit (PALL) and centrifuged at 8000 x *g* for 30 min at room temperature. Filter units were washed in 8 M urea diluted in 100 mM TEAB four times. Proteins were then dissolved in 5 M urea/100 mM TEAB and then digested in the filter unit with 0.5 μg Lys-C (Wako) at 37 °C for 1 h. Samples were then diluted to 1 M urea in 100 mM TEAB and then digested again with 0.5 μg of sequencing-grade trypsin (Promega) at 37 °C overnight. Digested samples were collected from the filter by centrifugation and then acidified to approximately 1% trifluoroacetic acid (TFA) and then desalted on a C18 solid phase extraction cartridge (GL Sciences). Peptides were eluted from the column with 80% acetonitrile/1% TFA and then dried in a SpeedVac concentrator (ThermoScientific).

Tryptic peptides were analysed in a nano-flow LC-MS/MS system using a Q Exactive HF mass spectrometer coupled with an online UltiMate 3000 Rapid Separation LC (Thermo Fisher Scientific) and an HTC PAL sample injector (CTC Analytics). Each sample was processed in data-dependent analysis (DDA) mode and all mass spectra were analysed with Proteome Discoverer v2.2 (ThermoFisher Scientific) using a human Swiss-Prot database incorporated with tau-441 sequence. This was followed by the label-free quantification of the identified peptides in each sample. To account for the variation in general tau abundance between individuals, the normalised abundance (%) of each peptide within an individual sample was calculated by dividing the amount of measured peptide by the sum of all tau peptides in the same sample multiplied by 100. The standardised abundance for each peptide was then used to allow comparison between samples. This was calculated using the following formula: (X - μ)/σ where X is the normalised abundance of a peptide within a given sample, μ is the mean of normalised peptide abundance across all samples and σ is the standard deviation of normalised peptide abundance across all samples. Changes in standardised abundance valued as the number of standard deviations above or below the mean could then be compared between AD, PSP and control cases.

### Size exclusion chromatography

Aliquots of human brain ‘whole homogenate’ samples as described above were centrifuged at 21,000 x *g* for 30 min at 4 °C. The supernatant was retained and used as input material for chromatography. Briefly, 250 μL of sample was loaded onto a Superdex 200 Increase G/L column connected to an Akta FPLC system (GE Healthcare). The chromatography buffer was PBS and 1 mL fractions were collected over 1.5 column volumes. Prior to use, the columns were calibrated with protein standards using the Gel Filtration HMW calibration kit (GE healthcare). To detect tau proteins, 75 μL of each fraction was removed and mixed with 25 μL 4x LDS sample buffer (Invitrogen) containing 10% beta-mercaptoethanol. Proteins were then resolved on 4–12% Bis-Tris Novex gels (Invitrogen) and transferred to Hybond nitrocellulose membranes (GE healthcare). Blots were blocked for 1 h at room temperature in blocking buffer: a 1:1 mixture of Odyssey blocking buffer (LI-COR) and Tris-buffered saline containing 0.1% Tween-20 (TBS-T). After blocking, blots were probed with primary antibodies: either 7G6 (0.5 μg/mL) or HT7 (Thermo Scientific, 0.2 μg/mL) diluted in blocking buffer. Blots were washed three times in TBS-T (10 min each wash) before incubation with an IRDye 700RD goat anti-mouse secondary antibody (LI-COR) diluted at 1:5000 in blocking buffer for 1 h at room temperature. The secondary antibody was removed and blots were washed four times in TBS-T and then a further two times in TBS without Tween-20. Blots were then scanned and fluorescent images acquired using an Odyssey LiCor CLx scanner with the Image Studio software (LI-COR).

## Results

### Generation and humanisation of antibodies

For generating antibodies, two peptide immunogens were designed to span sequences within R2 and R3 of the MTBR in 4R-tau that include the sequences essential for pathological seeding and aggregation (Fig. 1) [11, 77, 78]. Peptide 1 (19 amino-acids) was designed to exclusively target 4R-tau isoforms and incorporated 17 residues from R2, including the PHF6* (_275_VQIINK_280_) motif and two residues from the adjoining R1. Peptide 2 (19 amino-acids) spanned the R2-R3 junction with the adjoined _299_HVPGGGS_305_ and PHF6 (_306_VQIVYK_311_) sequences that together form metastable compact structures that modulate aggregation propensity [11]. An additional N-terminal cysteine was added to Peptide 2 for protein-coupling purposes. Peptides were conjugated to KLH to immunise *Mapt*-null mice in order to generate hybridomas. After ELISA screening and single-cell cloning, four antibody-producing hybridomas against Peptide 1 and eight against Peptide 2 were considered of interest for further study.

The affinities of the 12 antibodies for wild-type 2N4R-tau protein were initially measured by surface plasmon resonance (SPR) and the K_D_ values are given in Additional file 1: Table S1. We also initially determined selectivity of the antibodies to 3R- and 4R-tau by dot blot (Additional file 1: Table S1 and Additional file 1: Fig. S1A). Based on its exceptionally high affinity for wild-type recombinant tau expressed in *E.coli* (K_D_ = 52 pM), we selected clone 7G6, raised against Peptide 2 for further characterisation and subsequent humanisation. The dot blot showing the ability of 7G6 to bind decreasing amounts of 4R- or 3R- tau isoforms is shown in Additional file 1: Figure S1A.

The 7G6 antibody was humanised by grafting the complementarity-determining regions (CDRs) onto a human IgG1 backbone with IGHV3 and IGKV1 frameworks (Additional file 1: Figure S2). These frameworks, commonly represented in humans, have been used previously in FDA-approved therapeutic antibodies and are thought to present a lower risk of clinical immunogenicity. Also, a cysteine residue in CDR2 of the heavy chain, predicted to be solvent exposed and thus increasing the likelihood of post-translational modification (especially oxidation), was substituted to a serine residue. This substitution did not affect tau binding as assessed by ELISA (data not shown). The final humanised version of the 7G6 antibody was designated E2814.

To confirm that antibody characteristics and efficacy were maintained during the humanisation process, both 7G6 and E2814 antibodies were biotinylated and captured onto streptavidin-coated SPR chips. Full-length human wild-type 2N4R tau was then used as the analyte to determine antibody affinity. Table 1 shows that high affinity was retained during the humanisation process: 7G6 and E2814 had respective affinities of 64 pM and 88 pM. The slightly lower affinity of E2814 was a consequence of a slightly faster off-rate compared to the 7G6 antibody.

**Table 1:** Affinity determination of 7G6 and E2814 antibodies to wild type tau protein

Antibody	k_a_(1/Ms)	k_d_(1/s)	K_D_ (M)
7G6 (mouse)	3.11 × 10^6^	1.95 × 10^−4^	6.36 × 10^−11^
E2814 (human)	3.50 × 10^6^	3.07 × 10^− 4^	8.81 × 10^− 11^

Noting the comparable affinity of 7G6 for both 3R- and 4R-tau, we assessed this with the humanised E2814 using SPR. Using a concentration range (5, 10, 20, 40 and 80 nM) of cell-free expressed recombinant 2N4R and 2N3R tau as analytes, we showed dose-dependent binding of E2814 to both isoforms. E2814 retained strong affinity for 2N3R tau despite having one less binding site with the affinity being 0.37-fold lower when compared to 2N4R-tau (Additional file 1: Figure S1B).

### Characterisation of 7G6 and E2814: Epitope-mapping with tau peptide microarray

**Fig. 2:**
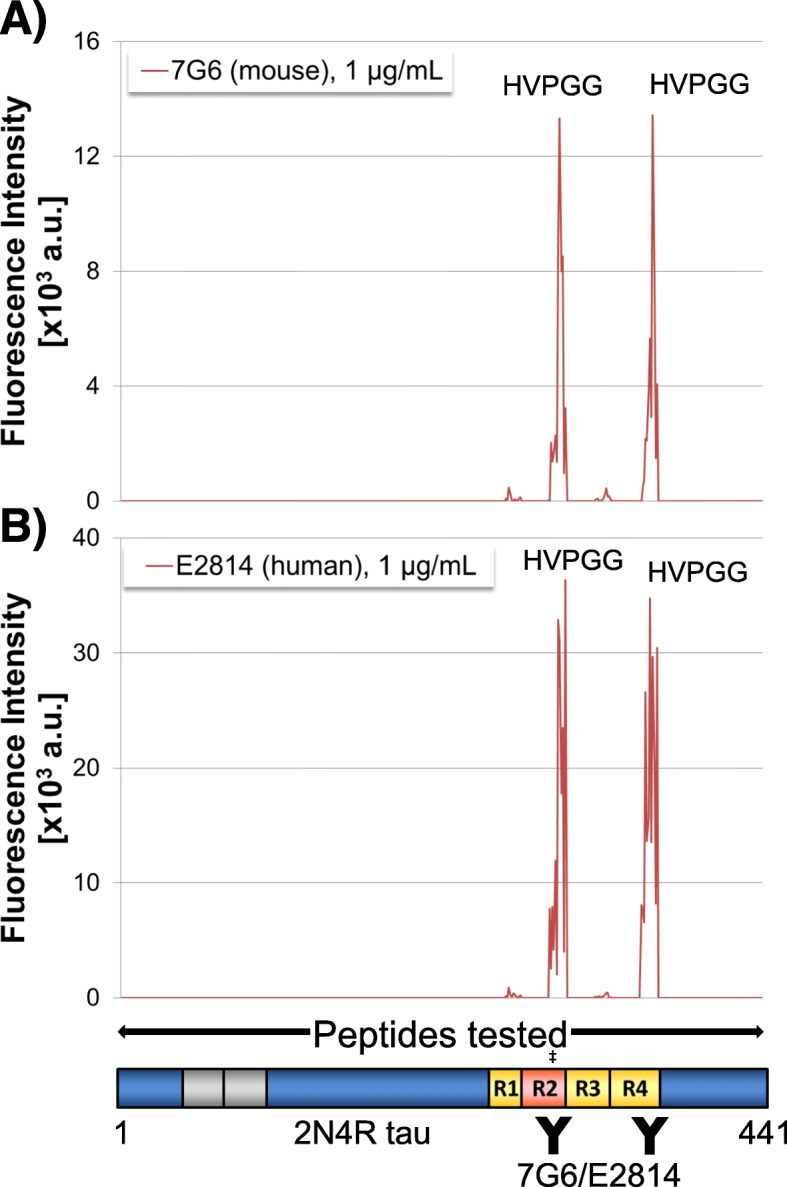
Fine epitope mapping of 7G6 and E2814 anti-tau antibodies. Overlapping peptides covering the full length wild type human 2N4R tau sequence were synthesised and printed onto glass chips. Each chip was then probed with either **a**) 7G6 (mouse) or **b**) E2814 (human) antibodies at 1 μg/mL. Bound antibodies were detected by the addition of fluorescently-labelled secondary antibodies and images captured using a LI-COR Odyssey machine. Fluorescent spots corresponding to antibody-bound peptides were quantified and intensity plots spanning the full tau sequence were generated for both murine 7G6 (**a**) and human E2814 (**b**) antibodies. Both antibodies bound peptides containing an HVPGG sequence as indicated in each panel

To identify the precise tau sequence recognised by 7G6 and E2814 antibodies, fine epitope mapping was carried out using PEPperCHIP® customised peptide microarrays consisting of overlapping peptide sequences covering the entire 2N4R-tau protein (PEPperPRINT GmbH, Heidelberg, Germany). Both 7G6 and E2814 bound equally to HVPGG motifs found in the second- (R2; amino-acids 299–303) and fourth (R4; amino acids 362–366) repeat in 2N4R-tau (Fig. 2). Hence, both antibodies are bi-epitopic for 4R-tau. However, as R2 is absent in 3R-tau, only one of the HVPGG binding epitopes is present. This helps explain the lower affinity of E2814 for 3R-tau as described above. Two lower intensity signals indicated trace binding to HQPGG at amino acid positions 268 to 272 in R1; and HKPGG at positions 330 to 334 in R3 of 2N4R-tau.

Human *MAPT* gene mutations resulting in substitution of the proline at positon 301 (P_301_) of 2N4R-tau with a serine or leucine, is a cause of the tau-variant form of familial frontotemporal dementia. Since the P301S/L mutations cause a greater propensity for tau to aggregate, this mutation is often used pre-clinically to enhance tau aggregation both in vitro and in transgenic mouse models of tauopathy. Noting that this residue resides in the HVPGG motif in R2, we tested whether the mutation would impair binding of 7G6 to the mutated protein. SPR analysis (Table 2) demonstrated that, although 7G6 retains high, sub-nanomolar binding affinity (448 pM) to 2N4R-tau with the P301S mutation, this was lower compared to wild-type 2N4R-tau (36.9 pM).

**Table 2:** Affinity determination of 7G6 antibody to wild type and P301S mutant tau protein

Antigen	k_a_(1/Ms)	k_d_(1/s)	K_D_ (M)
Wild type tau	1.65 × 10^6^	6.10 × 10^− 5^	3.69 × 10^− 11^
P301S tau	6.82 × 10^5^	3.06 × 10^− 4^	4.48 × 10^− 10^

To aid in interpretation of pre-clinical experiments utilising cellular and in vivo models expressing P301L/S mutant tau and to further investigate the binding requirements of 7G6, substitution analysis was performed using the peptide _1_KDNIKHVPGGGSVQI_15_ by systematically replacing each amino acid in the sequence with all 19 other amino acids (Additional file 1: Figure S3). Residues H_6_, P_8_ and G_9_ (H_299_, P_301_ and G_302_ in the full-length protein), were found to have no tolerance for substitution by any amino acid. These data would also imply that H_362_, P_364_ and G_365_ are essential at the second HVPGG binding motif in the fourth repeat (R4). Importantly, substitutions corresponding to the P301S or P301L mutations completely abolished binding in support of our observation that, like 3R-tau, 7G6 binding to the full-length recombinant P301S mutant protein was due to recognition of just the intact HVPGG epitope within the R4 domain. Minimal tolerance for substitution was observed at the G_10_ residue whereas a moderate tolerance at V_7_ was detected. Residues flanking the HVPGG core motif also exert minor influence of 7G6 binding to the peptides.

### Western Blot and Immunohistochemical validation

The specificity of 7G6 and E2814 antibodies were demonstrated by western blotting (Additional file 1: Figure S4). Tau proteins were detected in sarkosyl-insoluble and -soluble brain fractions from rTg4510 transgenic mice [67] that overexpress human mutant P301L 0N4R tau, sarkosyl-soluble brain fractions from wild type mice but immunoreactivity was completely absent in total lysate from *Mapt*-null mice (Additional file 1: Figure S4A). Immunoreactivity to different isoforms of tau could not be assessed in these mouse samples since only 4R-tau is expressed in adult mouse brains [55].

To further confirm binding and isoform selectivity, we immunoprecipitated tau from Alzheimer’s disease (AD) and Pick’s disease (PiD) brain lysates with E2814 and with IgG_1_ as an isotype control. By western blotting with a pan-tau antibody (Dako, K9JA) of the precipitated (and dephosphorylated) proteins, and the unbound protein in the flow through, we show effective capture of both 3R-tau and 4R-tau by E2814 (Additional file 1: Figure S4B). E2814 immunoprecipitated protein showed relatively equal ratios of 3R-tau and 4R-tau and, also, near complete depletion in the unbound fraction.

We then sought to determine whether E2814 and 7G6 could recognise pathological tau features in fixed, human brain sections. The antibodies were tested on sections obtained from AD, progressive supranuclear palsy (PSP) and PiD patients to ascertain if they recognise the pathological tau inclusions characterising these cases. In addition to the hallmark extracellular β-amyloid plaques, the AD brain is characterised by intraneuronal neurofibrillary tangles (NFTs) and neuropil threads, with the occasional astrocytic plaques consisting of insoluble aggregates of both 3R- and 4R-tau isoforms. In PSP brain, the more globose NFTs consist almost exclusively of 4R-tau [16] and, as with all 4R-tauopathies, also harbour 4R-tau glial inclusions in the form of tufted astrocytes (TAs) and oligodendroglial coiled bodies (CBs). Conversely, Pick bodies (PBs) in PiD contain only 3R-tau [16, 17, 23].

**Fig. 3:**
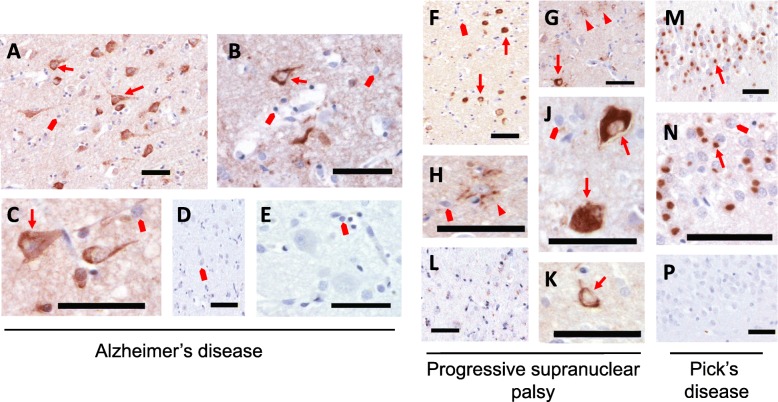
Immunohistochemical staining of tauopathy sections with E2814. **a**-**c**: Frontal cortex from AD brain sections stained with 0.5 μg/mL E2814 showing robust labelling of neurofibrillary tangles (arrows). Nuclei stained in blue (quad arrow); **d**, **e**: Control staining of AD brain sections with human IgG also at 0.5 μg/mL. **f**-**h**,**j**,**k**: Frontal cortex from a PSP patient stained with E2814 (0.2 μg/mL) showing widespread staining of neurofibrillary tangles (arrows) and glial inclusions, including tufted astrocytes (G,H; arrowheads) and oligodendroglial coiled body (**k**; arrow). **l**: Control staining of a PSP section with human IgG (0.33 μg/mL). Non-specific brown colouration is due to lipofuscin. Nuclei are stained in blue (quad arrows); **m**,**n**: Hippocampus from a PiD patient stained with E2814 (0.2 μg/mL) showing strong staining of Pick bodies (arrows). **p**: Control staining of PiD brain section with human IgG (0.33 μg/mL). Nuclei are stained in blue (quad arrows). Scale bars = 50 μm

We first optimised staining with different dilutions of the 7G6 and E2814 antibodies (1/2000 (0.5 μg/mL), 1/3000 (0.33 μg/mL) and 1/5000 (0.2 μg/mL)) in order to specifically label the pathological inclusions, where tau protein is enriched, over the basal cytoplasmic tau levels. With all three clinical cases, both 7G6 and E2814 robustly stained all the cognate pathological tau inclusions (Fig. 3 (E2814) and Additional file 1: Figure S5 (7G6)) including NFTs and neuropil threads in AD (Fig. 3 a-c) and NFTs as well as the TAs and CBs in PSP (Fig. 3 f-k). Robust staining of the PBs in the PiD hippocampal granule cells (Fig. 3 m and n), confirms strong binding despite the presence of only one of the two HVPGG binding motifs in 3R-tau-dominant PiD pathology [16, 17, 23]. Although E2814 can clearly bind the end-stage fibrillar tau structures in post mortem disease brain, it should be noted that the antibody does not selectively bind to defined pathological conformers of tau or phospho-tau proteins. This is demonstrated by Additional file 1: Figure S6 where, in areas adjacent to those with tau pathology, E2814 labels non-fibrillar tau in neuronal cytoplasm as well as neuropil, when compared to the human IgG_1_ control.

### Immunogold electron microscopy labelling of pathological tau fibrils

**Fig. 4:**
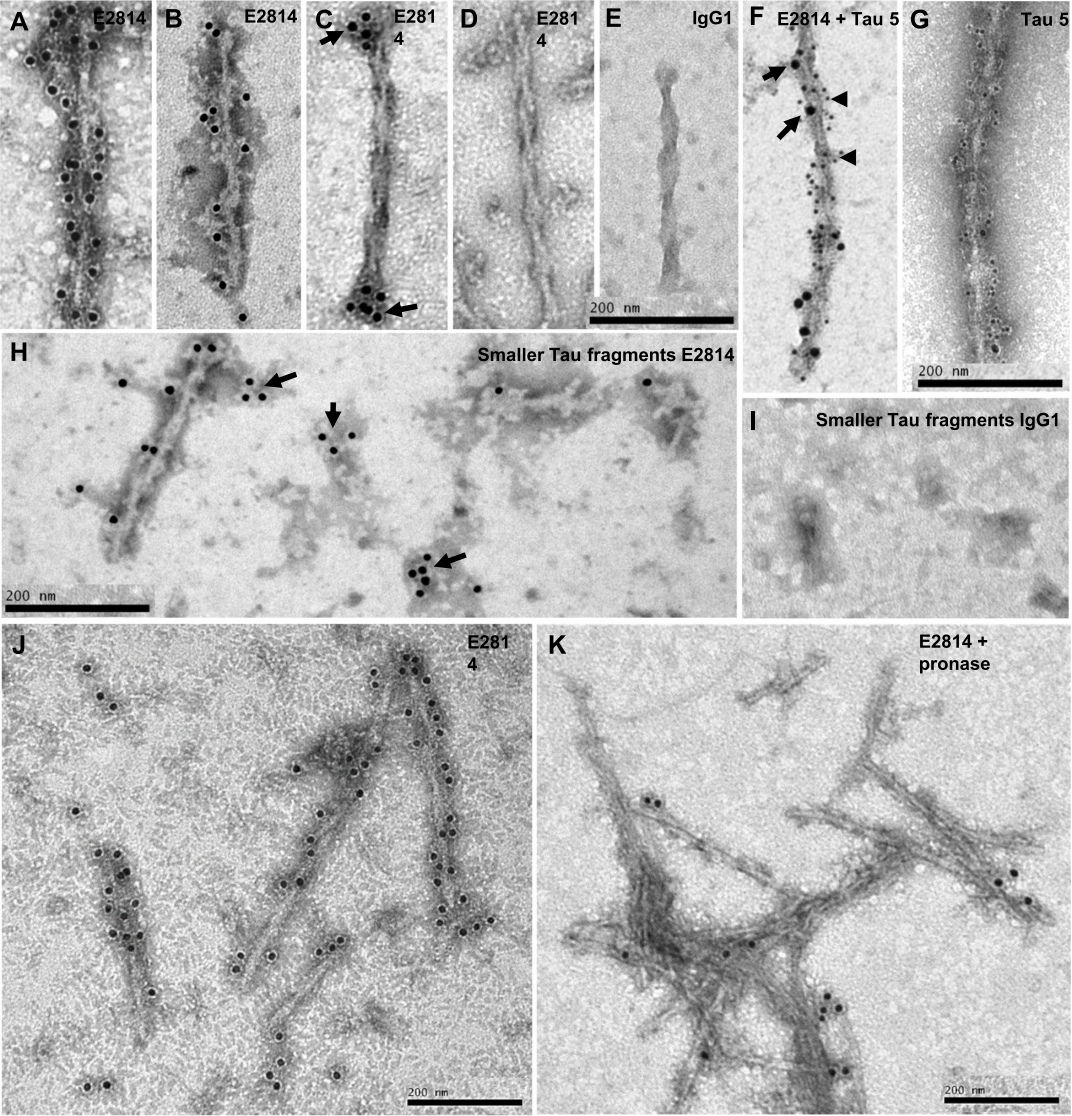
Immunogold labelling of tau filaments from human AD brain. Representative electron microscope images of tau fibrils isolated from the sarkosyl-insoluble fraction of AD patient frontal cortex. E2814 or IgG1 isotype control were used at 10 μg/mL. Tau 5 antibody was used at 0.4 μg/mL. Bound antibody was detected following addition of an anti-human 12 nm gold conjugated antibody at 1:25 dilution or an anti-mouse 6 nm gold conjugated antibody at 1:25 dilution (for Tau 5). **a**, **b**: E2814 could bind the entire length of many tau fibrils. In some paired helical filaments (PHFs) E2814 binding was limited to the ends (arrows) of the fibrils (**c**) or was completely absent (**d**). **f**: E2814 (arrows) and the commercially available Tau 5 antibody (arrowheads) co-stain tau fibrils, providing proof of E2814 specificity to tau fibrils. **g**: Tau 5 binds to the entire length of tau fibrils. **h**: E2814 specifically binds to smaller structures on the EM grids that may represent tau fibril fragments or tau oligomers (arrows). The IgG1 control antibody did not bind to filbrils (**e**), or smaller fragments (**f**). **j**,**k**). Pre-treatment of AD fibrils with 0.4 mg/ml pronase removed the fuzzy coat of PHFs and SFs, leaving the structured core intact with E2814 staining retained only at some fibril ends (Scale bars = 200 nm)

To further characterise E2814 and 7G6 antibodies and to confirm that the HVPGG epitope was retained and accessible in pathological tau fibrils, antibody binding was determined by immunogold labelling. Tau fibrils and other structures in the sarkosyl-insoluble fraction of brain from an AD patient (Braak VI) were labelled with E2814 or a human IgG_1_ control antibody as described under Additional file 1: Supplementary Materials and Methods. We used a series of antibody dilutions (10–0.05 μg/mL) to determine optimal labelling. Saturation of possible binding sites at these optimal concentrations were determined by incubation with a very high concentration of antibody (100 μg/mL) which showed similar labelling patterns and densities compared to 10 μg/mL (Additional file 1: Figure S8 F + G). Furthermore, specificity of binding was demonstrated by double labelling of the AD tau fibrils with E2814 and Tau-5 antibody (Fig. 4 f) and complete absence of immunogold labelling with the IgG control antibody (Fig. 4 e + i).

The E2814 antibody binds to the entire length of many filaments. In some PHFs however, the binding was restricted to their ends or sometimes absent (Fig. 4 a-d). E2814 was also able to recognise smaller tau fragments that could either be due to shearing during the tissue processing or immature fibrils and perhaps even oligomers (Fig. 4 h), the latter suggested to be the transmissible pathological tau species in human disease [30]. The same binding pattern was also observed with the murine 7G6 antibody (Additional file 1: Figure S7). Treatment of the fibrils with pronase to remove the fuzzy coat abolished E2814 binding along the length of the fibrils (Fig. 4 J and K), suggesting that E2814 binds mainly to the R2 epitope that resides in the the fuzzy coat around the AD tau fibril [26]. Only end-binding of fibrils remained after pronase treatment, suggesting that the R4 epitope may be exposed at fibril ends.

In addition to AD fibrils, E2814 robustly decorated tau fibrils isolated from the frontal cortex of a frontotemporal dementia (FTLD-tau) patient with the *MAPT* missense mutation R406W (Additional file 1: Figure S8A), and another patient carrying the *MAPT* Δ280K deletion (Additional file 1: Figure S8B). Occasional labelling was observed on tau fibrils isolated from the frontal cortex of a FTLD-tau patient with the IVS10 + 16 mutation that leads to increased incorporation of the exon 10 (Additional file 1: Figure S8C). In PSP, E2814 did not bind along the length of isolated fibrils but was seen on some smaller structures (Additional file 1: Figure S8D). In fibrils isolated from the frontal cortex of a Pick’s disease patient, binding was only observed at the filament ends (Additional file 1: Figure S8E). These data illustrate that the structures of mature tau fibrils differ between the tauopathies as well as between mutation carriers.

In support of our observation that E2814 can bind to the HVPGG epitope in pathological tau fibrils isolated from tauopathy brains, we also demonstrated binding to tau aggregates in solution. By immunoprecipitation, we showed that E2814 effectively binds to and depletes seed-competent K18 fibrils (truncated tau; amino acids 244–372) (Fig. 6 a and b) and 7G6 recognises pre-formed recombinant 2N4R P301S tau fibrils (Additional file 1: Figure S11).

### Inhibition of tau aggregation and seeding

**Fig. 5:**
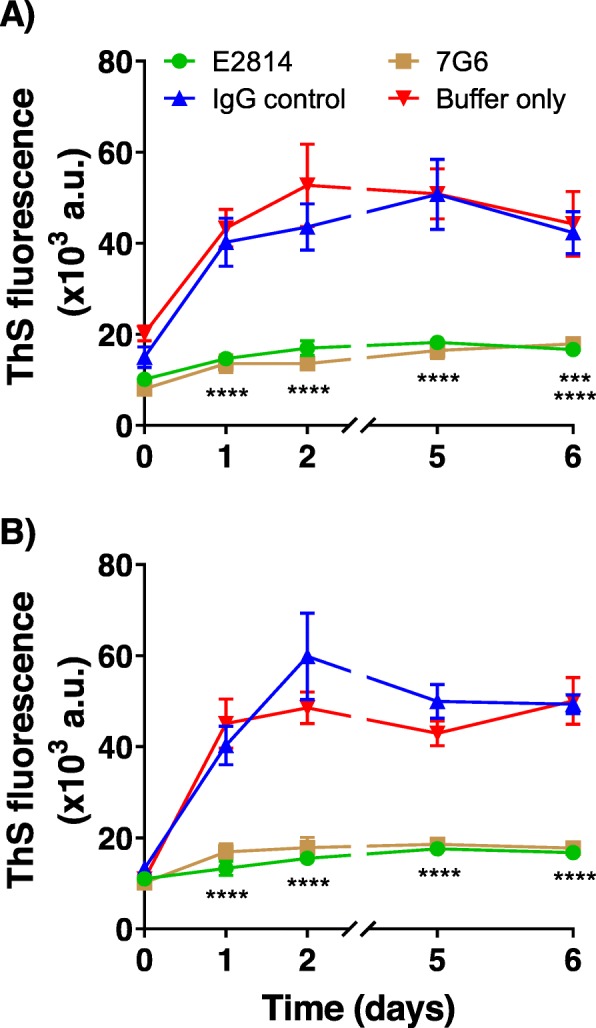
Inhibition of tau aggregation in vitro. Recombinant wild-type (**a**) or P301S mutant (**b**) tau at 12 μM was induced to aggregate in vitro with addition of heparin in the absence or presence of either 7G6, E2814 or control IgG_1_ antibodies at a concentration of 8.3 μM. Over a time course of 6 days, samples of the reaction mixture were removed and incubated with Thioflavin S (ThS) and fluorescence was measured to detect aggregated tau. Data shown represent six independent experiments for each protein. A two-way ANOVA statistical analysis was performed followed by a Dunnett’s test. **** *p* ≤ 0.0001 7G6 or E2814 versus IgG, *** *p* ≤ 0.001 7G6 vs IgG for wild type protein only. Values represent mean ± SEM

Next, we evaluated the biological functionality of 7G6 and E2814 antibodies. Recombinant 2N4R tau protein was aggregated in vitro by co-incubation with heparin over several days and aggregates were revealed and quantified by Thioflavin S (ThS) binding. Heparin-induced aggregation was carried out with incubation of approximately equimolar amounts of wild-type or 2N4R P301S mutant recombinant tau in the absence (buffer alone) or presence of either 7G6, E2814, or control IgG. Both 7G6 and E2814 showed a strong and significant inhibitory effect on tau aggregation (Fig. 5), suggesting that, if aggregation-competent tau is accessible to and bound by E2814, the antibody may have the ability to prevent further tau aggregation in a disease setting.

**Fig. 6:**
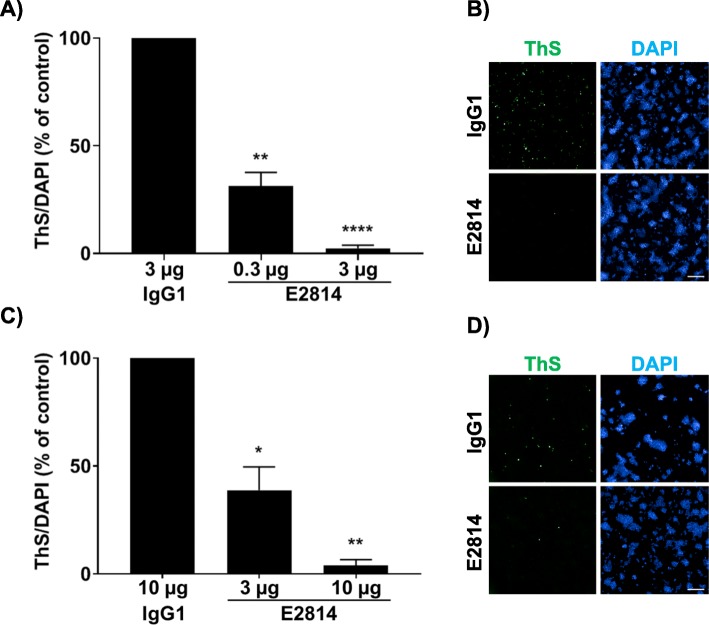
Immunodepletion of tau seeds with E2814 reduces intracellular tau deposition. E2814 antibody or human IgG_1_ control at specified concentrations were used to immunodeplete K18 fibrils (**a**,**b**) or full length P301S tau monomer (**c**,**d**) seeds. Treated samples were added to HEK293 cells overexpressing P301S mutant 0N4R tau. Intracellular tau deposition was measured by addition of Thioflavin S (ThS) and cells were counterstained with DAPI to visualise the nuclei. The percentage of ThS/DAPI relative to IgG_1_ control is plotted (IgG_1_ = 100% seeding effect). Values represent the mean ± SEM from four (**a**) and three (**c**) independent experiments. Data were analysed by one way ANOVA followed by Dunnett’s test. *P < 0.05, **P < 0.005, **** < 0.0001. Representative images of ThS and DAPI staining for K18 fibril (B, 3 μg antibody) and P301S monomer (**d**, 10 μg antibody) immunodepleted seeds are shown. Scale bars = 200 μm

It has been reported that the MTBR region of tau is necessary to form seeds that propagate tau pathology in humans [22]. To verify these findings, a cell-based assay of tau deposition was established. Fibrillar or monomeric tau proteins were added to HEK293 cells overexpressing 0N4R P301S tau, and after 48 h intracellular tau deposition was detected by staining the cells with Thioflavin S. Recombinant tau proteins containing the MTBR but not those without the MTBR were able to induce intracellular tau deposition (Additional file 1: Figure S9), thus confirming previous reports. The most effective MTBR-containing tau seeds tested in this assay were K18 fibrils (truncated tau; amino acids 244–372) and the full-length 2N4R P301S monomer. Next, to determine whether E2814 had the ability to recognise such seeds in their native form, the antibody or an IgG control was used to immunodeplete both K18 tau fibrils and 2N4R P301S tau monomer prior to cell addition. Intracellular tau deposition was reduced for both types of seed in a dose-dependent manner when depleted with the E2814 antibody compared to IgG control (Fig. 6). These data raise the possibility that pathological MTBR-containing tau seeds in human brain could be sequestered and masked by E2814.

**Fig. 7:**
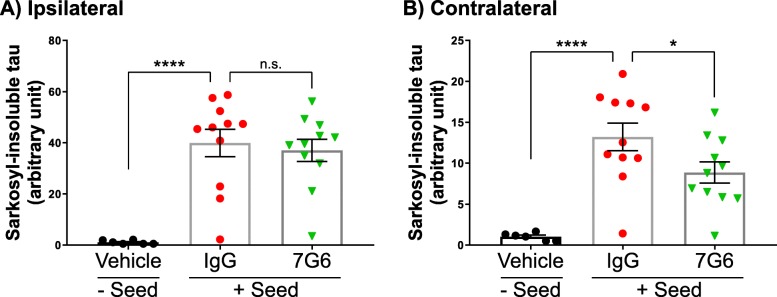
7G6 efficacy in an in vivo model of tau seeding and transmission. Full-length P301S tau seeds were injected into the left hippocampus of P301S transgenic mice pretreated with either IgG control (*n* = 11) or 7G6 antibody (*n* = 11) at a dose of 40 mg/kg i.p. Animals then received the same dose of antibody or vehicle once per week for a further 3 weeks until sacrifice. Vehicle-treated animals receiving no seed (*n* = 6) were included as an additional control group. Both hippocampi from each brain were extracted and separately treated with sarkosyl. Tau levels were then quantified in the sarkosyl-insoluble fraction from each sample by western blot and plotted for either the ipsilateral (**a**) or contralateral (**b**) hippocampi for each animal. Data are expressed as mean ± SEM and further analysed using a one-way ANOVA followed by Fisher’s LSD test. **** *p* ≤ 0.0001 IgG versus no seed control, * *p* ≤ 0.05 7G6 versus IgG for contralateral hippocampus, n.s. not significant

To determine whether E2814 could potentially prevent transmission of pathological tau in the brain, the murine antibody 7G6 was tested in a pre-clinical in vivo model of tau transmission. The model requires full-length 2N4R P301S tau seeds to be injected into the hippocampus of 3 to 4 month old 0N4R P301S transgenic mice [3] backcrossed onto a C57/BL6 genetic background. At this age, the mice show no overt tau pathology in the brain as assessed by immunostaining for pathological tau species (data not shown). After 3 weeks, brain tissue from the ipsilateral and contralateral sides to injection were extracted and the levels of sarkosyl-insoluble tau quantified by western blot. To test antibody efficacy, 7G6 or an IgG control antibody was dosed peripherally (i.p.) at 40 mg/kg prior to seed administration and then once per week for a following 3 weeks. A group of animals that were dosed with vehicle but did not receive tau seed were included as controls. Following peripheral 7G6 treatment, a modest, yet significant reduction in sarkosyl-insoluble tau levels was observed in the contralateral hippocampus compared to the IgG control group (Fig. 7 b; Additional file 1: Figure S10). However, a significant effect in the corresponding ipsilateral tissue was not observed (Fig. 7 a; Additional file 1: Figure S10). The data suggest that 7G6 (and E2814) are capable of attenuating the transmission of pathological tau species in this in vivo model. To show that the 7G6 antibody had successfully crossed the blood-brain barrier, albeit in expected low amounts, antibody levels were measured in both plasma and CSF of treated animals (Additional file 1: Table S2). The mean plasma/CSF concentration ratio was 0.08% in this experiment.

### AD brain tau peptide mapping

**Fig. 8:**
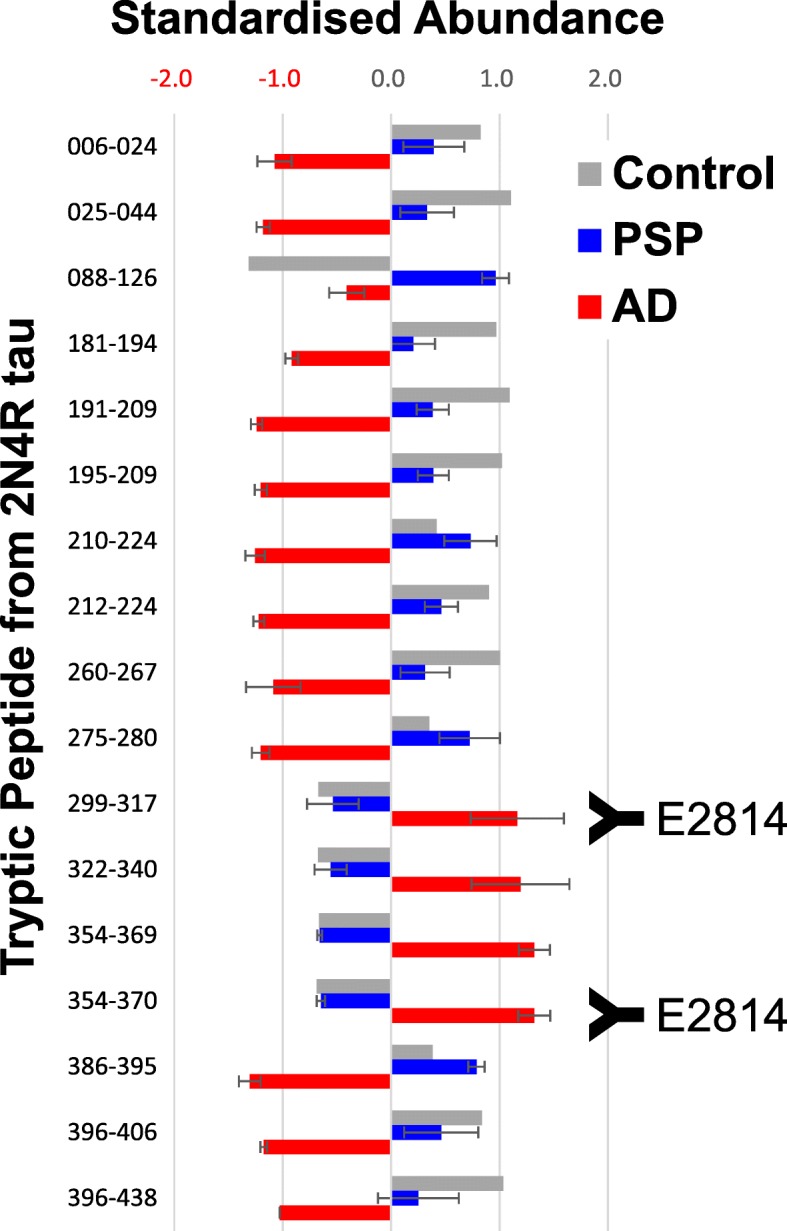
LC/MS analysis of tau peptide abundance in the insoluble fraction from human tauopathy brain. Insoluble fractions from AD (*n* = 3), PSP (*n* = 4) and control (*n* = 2) brain samples were prepared and digested with Lys-c and trypsin. Tryptic peptides were identified and quantified by LC/MS. The standardised abundance for each tau peptide was calculated (see Materials and Methods) and compared between AD, PSP and control brain samples. Peptides showing significant change between PSP and AD are plotted. Error bars for AD and PSP represent standard deviation

It is well documented that tau protein from human brain is proteolytically cleaved at numerous positions along its length [38, 51, 79] and suggested that this truncation may play an important role in AD pathology [62]. Many of the resulting tau fragments contain the MTBR but it is not possible to state currently, whether in human brain, full-length tau seeds or MTBR-containing fragments are responsible for propagation of pathology. To gain a deeper understanding of whether the regions of tau containing the E2814 epitopes alter in disease, a mass spectrometry technique was employed to compare relative levels of tau tryptic peptides spanning the whole tau protein in the insoluble fractions from different tauopathy and control brains. Following relative quantification, several tryptic peptides across the protein showed significant changes between AD, PSP and control samples. Most notably, peptides 299–317, 322–340, 354–369 and 354–370 were enriched in AD samples compared to PSP or control (Fig. 8). These peptides, containing both E2814 epitopes, in the tau MTBR are within the C-terminal half of R2 to the end of R4. Conversely, other peptides showing significant change between AD and PSP were either contained N-terminal to 299–317 or C-terminal to 354–370. The data demonstrate that in the insoluble fraction of AD brain compared to PSP or control, although using low numbers of samples, an accumulation of the tau MTBR containing both E2814 epitopes is evident. This raises the possibility that the C-terminal half of the tau MTBR may be more important in AD pathogenesis compared to other tauopathies.

**Fig. 9:**
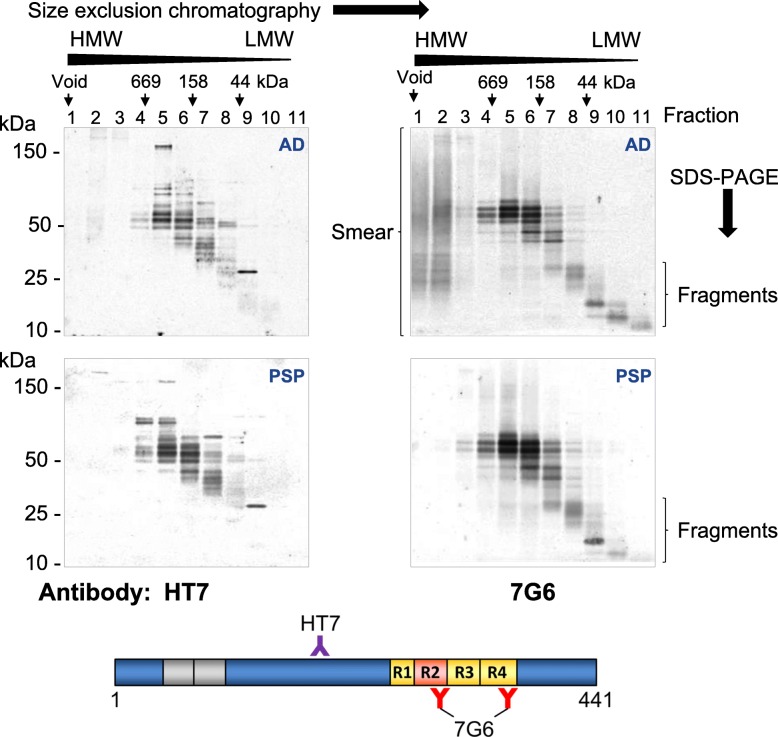
Size exclusion chromatography of tauopathy brain samples. Human brain homogenates from AD (upper panels) or PSP (lower panels) patients were loaded onto a Superdex 200 Increase GL size exclusion column in PBS and 1 mL fractions were collected. Samples from each fraction were resolved by SDS-PAGE and tau proteins were detected by western blotting using antibodies raised against two different parts of the full length protein: HT7 (0.2 μg/mL) or 7G6 (0.5 μg/mL) as indicated in the left or right panels, respectively. Images shown are representative from four different AD and six PSP brains

To investigate further the fragments of tau in tauopathy brain recognised by the 7G6 antibody, human brain homogenates were analysed by size exclusion chromatography. Brain homogenate was loaded onto the column and fractions were then collected from the first eleven fractions after the column void volume. Each fraction was analysed by western blotting using 7G6 or the anti-tau antibody HT7 which recognises the mid-domain of tau at amino acids 159–163 (Fig. 9). When analysing all AD samples, tau fragments resolving as a smear were only detectable in the high molecular weight fractions when revealed by MTBR-recognising 7G6 antibody but not with the mid-domain HT7 antibody. Similar data have been reported previously using other antibodies [58]. These observations suggest that multiple truncated tau species containing the MTBR are found in the higher molecular weight fractions and the majority of these tau species do not contain the mid-domain, HT7-binding portion of the protein. The tau smears in the higher molecular weight fractions detectable with the 7G6 antibody were only apparent in samples from AD brain and neither PSP brain (Fig. 9) nor control brain (data not shown). Both antibodies detected abundant bands in fractions 4 to 7, most likely to be fully intact tau isoforms. However, further, discrete truncation of tau was clearly visible in the lower molecular weight fractions 8 to 11 with more truncated tau species containing either the whole or part of the MTBR identified on the basis of strong immunoreactivity being observed with 7G6 but not HT7. A distinctive, discrete tight band in fraction 9 was clearly detected with HT7 but not the 7G6 antibody, suggesting this to be a tau fragment lacking the 7G6 epitopes within the MTBR. The low molecular weight tau species were detectable in all homogenates tested but the biological function, if any, of such fragments requires further investigation. If accessible, it is anticipated that E2814 binding to a variety of MTBR-containing tau species could prove therapeutically beneficial by either direct neutralisation or enhancing their clearance.

## Discussion

Therapeutic agents targeting tau proteins have emerged as a new strategy to alter disease progression in a number of tauopathies, including Alzheimer’s disease (AD). Increasing evidence suggests that tau pathology progressively spreads throughout the brain during disease course and contributes to the neuronal cell compromise and death ultimately responsible for onset and progression of symptoms. Early reports of tau pathology in AD Braak staging [8] described initial deposition in the entorhinal cortex followed by propagation to the limbic regions and beyond, giving strong credence to the notion that tau pathology spreads along connected neuronal pathways. It is also suggested that the spread of pathology occurs through extracellular pathological tau “seeds” that are transmitted from neuron to neuron [1]. The precise molecular nature of the seeds are unknown but this mechanism may provide several opportunities for therapeutic intervention, including passive immunization with appropriate monoclonal antibodies.

Here, we describe E2814 as a unique, high-affinity, bi-epitopic humanised IgG_1_ antibody for possible therapeutic application in AD and other tauopathies. Our initial strategy was to raise antibody candidates recognising regions within the tau MTBR that incorporate key sequences essential for the formation of pathological seeds and initiation of aggregation [22]. E2814, and its murine counterpart 7G6, bind to sequence epitope HVPGG present twice in tau isoforms with four microtubule-binding repeats (4R-tau; HVPGG is present within R2 and R4) and once in three-repeat (3R-tau) isoforms (R4 only). By binding to HVPGG, both antibodies effectively inhibit 4R-tau aggregation in vitro. In immunohistochemical staining of human brain tissue, the antibodies recognised mature pathological tau structures such as neurofibrillary tangles in AD and progressive supranuclear palsy (PSP) as well as Pick bodies in Pick’s disease (PiD), the latter a tauopathy whose inclusions contain only 3R-tau isoforms. This confirms the presence of the MTBR, containing the HVPGG in the inclusions. However, pathological phosphorylation- or conformation-specific antibodies such as AT8 [32] or MC1 [46], respectively, E2814 is not selective for pathological tau per se. Instead, by binding to the HVPGG epitope(s), E2814 could not only intervene in the misfolding of tau and formation of seeds but also facilitate their clearance.

Noting that the most widely used pre-clinical models of tauopathy employ cDNA-based transgenes overexpressing P301S or P301L variants whereby the mutation lies in the middle of the R2 HVPGG motif, it was of concern that weakened binding to tau with those mutations could limit experimental outcomes. Indeed, binding of 7G6 completely relied on the presence of the P301 residue since substitution by any other amino acid completely abolished binding (Additional file 1: Figure S3). However, even with just one HVPGG epitope in R4 remaining, 7G6 retained high-affinity binding (K_D_ = 448 pM) to 2N4R P301S tau but this was less when compared to the wild-type protein (36.9 pM). Both 7G6 and E2814 were equally effective in preventing in vitro heparin-induced aggregation of recombinant wild-type- and P301S tau (Fig. 5) despite loss of one epitope in the mutant protein. Furthermore, immunodepletion of seed-competent K18 peptide (repeat region only of wild-type 4R-tau) or recombinant 0N4R P301S monomer with E2814, resulted in effective removal of seeding activity in cells overexpressing 0N4R P301S tau. These data suggest that high-affinity binding of E2814 to the second HVPGG motif in R4 is sufficient to prevent seeded pathological spread and aggregation by masking the necessary conformational interfaces. As with binding to 0N4R P301S tau, we have also demonstrated that 7G6 and E2814 effectively bind to 3R-tau isoforms and expect therefore that these antibodies will also prevent aggregation of 3R-tau.

**Fig. 10:**
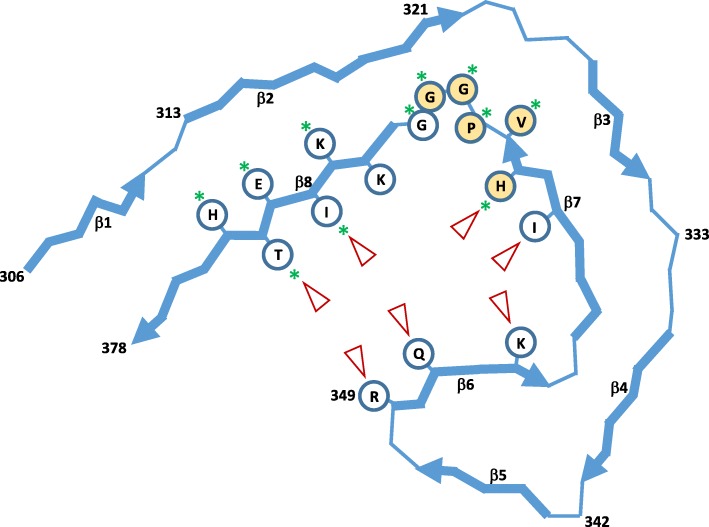
AD tau protofibril structure (modified from [26])**:** Schematic representation of protein backbone of the R3 + R4 protofibril unit of AD tau paired-helical filaments and straight filaments, with selected amino acid side chains. The numbering of amino acid positions is based on the 2N4R-tau isoform (NP_005901). Antiparallel β-strands are indicated by thick arrows. In AD, the protofibril adopts a compact C-shaped structure. The E2814 HVPGG binding motif (362-366; yellow) forms the tight bend between β_7_ and β_8_. Red arrowheads indicate predicted strong contact points to heparin [53] resulting in heparin-mediated aggregation by compaction and stabilization of the AD protofibril. Green stars indicate sequences that interact with azure A and azure B, monodemethylated derivatives of methylene blue (MB), which have anti-aggregation effects on tau by preventing fibril formation and retaining tau in monomeric form [2]

The conversion of highly-soluble tau into its seed-competent, aggregation prone form involves the short hydrophobic domains of the MTBR normally responsible for binding to microtubules [48]. New structural information has also emerged that has given further insight into the mechanism of pathological tau species formation to strengthen the rationale to further investigate E2814. For example, recent work has demonstrated that conformational conversion to seed competent tau is caused by unmasking the PHF6 motifs (_275_VQIINK_280_/_306_VQIVYK_311_), rendering them more accessible to illicit intermolecular seeding interactions and aggregation [57]. In soluble tau, these motifs are normally masked by adopting compact β-hairpin pairings with flanking sequences [57] that include the R2 HVPGG motif [11]. Furthermore, disease-causing missense mutations in the flanking N-terminal region, including those in the R2 HVPGG sequence such as P301L/S [10, 12, 19, 43, 66, 73, 74], significantly increase the aggregation propensity of tau by destabilising the β-hairpin to cause a more extended conformation of the region exposing the PHF6 motifs [11]. This allows cross-β sheet rearrangements and interactions resulting in highly compact and stable protofibril structures that self-assemble into the characteristic end-stage filaments. Protofibrils from different tauopathies also adopt unique characteristic folds [23, 24, 26]. In AD protofilaments for example, the HVPGG binding motif in R4 is essential for formation of the inner bend and compaction of the C-shaped protofibril but would be predicted as inaccessible to E2814 in structurally established protofibrils (Fig. 10). However, the HVPGG motif in R2 is not part of the compact protofilament, and is therefore predicted to be more accessible to both E2814 and 7G6 antibodies.

Considering these structural insights, we surmise that E2814 may exert an effect by binding to and stabilising the accessible HVPGG motifs in the β-hairpin pairings that mask the PHF6 motifs in soluble tau, thus preventing conformational conversion to seed-competent tau. E2814 binding at either R2 or R4 sites could also sterically prevent the intermolecular seeding interactions. We have shown here that E2814 retains efficacy in preventing heparin-mediated aggregation of P301S indicating that intervention at the R4 HVPGG site is sufficient. Although the R4 HVPGG motif is predicted to be inaccessible in the structurally mature AD protofibril, it is possible that binding by E2814 at earlier stages of conformational conversion and misfolding could prevent formation and compaction of the protofibril and subsequent fibril assembly.

To further illustrate the importance of the E2814 binding sites, heparin, a glycosaminoglycan that co-exists with tau in neurons with neurofibrillary pathology [63], and small molecule aggregation inhibitors can be used as examples. Heparin facilitates the aggregation of tau by exposing the PHF6 hexapeptide motifs [21] and, in accordance with the AD protofibril structure, is predicted to interact with amino acid side chains lining the groove of the C-shaped core to mediate folding and stabilisation. One of those amino acids is the H_362_ residue in the R4 HVPGG (362-366) motif (Fig. 10) [53]. Conversely, small molecule tau aggregation inhibitors, azure A and azure B, also interact with _362_HVPGGG_367_ as well as _371_IETH_374_ [2] contained within the R4 region. Given that E2814 could inhibit P301S tau aggregation in vitro it is plausible that binding to the R4 HVPGG is sufficient to sterically prevent formation and maturation of tau fibrils in AD brain.

Although the mechanism of tau seeding and propagation of pathology is poorly understood, it is now widely accepted that pathological tau variants (seeds) escape affected neurons and enter neighbouring, or synaptically-connected neurons [27]. It has been suggested that the transmissible seeds could either be high molecular weight assemblies [76], non-fibrillar oligomeric tau species [52] or, seed-competent conformations of monomeric tau [57]. In addition to inhibiting further aggregation, binding of MTBR-containing extracellular seed-competent tau species may lead to prevention of neuronal tau uptake [81]. Since E2814 is an IgG_1_ antibody with retained effector function, it is also possible that once bound to extracellular seed-competent tau species, microglial cells would be stimulated to accelerate clearance of the unwanted tau conformers in an antibody-dependent manner. Such clearance effects have previously been demonstrated in the clinic for human IgG_1_ antibodies targetting amyloid in AD patients [71]. Another potential clearance mechanism could be through the ubiquitously expressed cytosolic F_c_ receptor, tripartite motif protein 21 (TRIM21), that has been reported to mediate the degradation of antibody-bound tau seeds entering the cell [54].

To test the effect of 7G6 in vivo*,* we unilaterally injected fibrillar P301S mutant 2N4R-tau into the left hippocampus of P301S transgenic mice [3] and treated the animals systemically with 7G6 antibody or a mouse IgG control. After 3 weeks, we observed attenuated levels of sarkosyl-insoluble tau in the contralateral hippocampus in 7G6-treated mice compared with control-treated mice but no differences were demonstrated in the injected half of the brain (Fig. 7). Despite the limitations of these data, e.g. reliance on the P301S mutation which may underestimate antibody efficacy due to weakening of binding at the R2 site, they do support the notion that the 7G6 antibody reduces seeded propagation of tau aggregation in the brain by being able to target conformational intermediates. This supports our hypothesis, at least under the conditions of this model, that the humanised form of 7G6 (E2814) can target the tau species involved in transmission that lead to further tau seeding and aggregation.

A number of reports have also demonstrated that truncated tau species are important upstream contributors to pathological aggregation of tau [4, 28, 36, 37, 42, 59, 65] and that they can also form the pathological seeds [15, 62, 83]. Interestingly, CSF tau from AD and PSP patients have higher levels of tau fragments from the amino-terminus and central region of the protein but are depleted of fragments containing the MTBR [7, 56]. Perhaps, selective sequestration of MTBR fragments occurs in brain pathological inclusions. As an adjunct in the assessment of the potential therapeutic efficacy of E2814, we investigated the abundance of its target MTBR domains in insoluble, pathological tau fractions from AD and PSP brains as well as in brain homogenates. Mass spectrometric (MS) analysis of tryptic peptides from insoluble brain fractions revealed a particular enrichment of peptides from the MTBR domain in the samples studied from AD compared to PSP brain and control brain. By contrast, an accumulation of tau peptides from the amino- and carboxy-terminal as well as mid-domains of tau were apparent in samples of PSP brain and control brain compared with AD (Fig. 8). The more abundant MTBR domain peptides in AD span amino-acid residues 299–370 to include both E2814 binding motifs and the entire R3 and R4 domains that form the stable AD protofibril core. This specific over-represenation, compared to PSP and controls, suggests that the stable core AD protofibril is protected from endogenous proteolytic cleavage whether physiological or associated with pathological aggregation. Nevertheless, in the samples analysed, there are clear differences in the accumulation of MTBR-containing tau species in AD brain compared to PSP or control.

Although the protofibril structures from 3R−/4R-tauopathies (AD and CTE) and a 3R-tauopathy (PiD) were resolved by cryo electron microscopy (Cryo-EM) [23, 25, 26], it had previously been a challenge to isolate sufficient sarkosyl-insoluble fibrillar tau from 4R-tauopathies (e.g. PSP, CBD and argyrophilic grain disease). Recently however, the core CBD 4R-tau protofibril structure has now also been reported [82]. In our hands, normalised yields of insoluble tau from 4R-tauopathies are considerably lower (data not shown), suggesting less stable fibril structure with greater susceptibility to proteolytic degradation. This was evident when we compared size-fractionated tau from brain homogenates where we observed an absence of higher order 7G6-positive tau assemblies in PSP, compared to samples from AD (Fig. 9). Interestingly, the smear seen in the higher molecular weight fractions of AD brains (Fig. 9; Fractions 1 and 2) were labelled by 7G6, but not by HT7, an antibody that recognises the mid-region of tau suggesting excessive tau proteolysis had occurred but the MTBR region was retained in higher order assemblies. These data accord with similar findings of tau analysis in sarkosyl-insoluble brain fractions reported elsewhere using alternative antibodies [58]. It is currently unclear whether the accumulation of cleaved tau containing the MTBR forms the intracellular fibril core in AD, or is representative of the extracellular ‘ghost tangles’ that remain once the cell expires. Interestingly, we also see a consistent, discrete banding pattern between AD and PSP samples for the lower molecular weight tau fragments that are immunoreactive for either HT7 or 7G6 (Fig. 9). This reiterates that tau truncation is perhaps not a random process, but rather by highly specific proteolytic cleavages and non-enzymatic fragmentation [83]. For example, the discrete band identified at around 25 kDa (Fig. 9: Fraction 9) labelled only with HT7 suggests that it lacks the MTBR whereas smaller (< 25 kDa) fragments contain the MTBR.

Importantly, our combined immunohistochemistry, immunogold-EM, immunoprecipitation and western blot analyses demonstrate that E2814, and its murine parent antibody 7G6, recognise human brain tau (full-length or fragments) and thus could intervene in and prevent the crucial conformational folding of the MTBR that leads to the genesis of pathological seeding species. This would ultimately prevent the tight folding and maturation of the AD tau fibrils.

## Conclusions

We have described E2814, a humanised IgG_1_ antibody for passive immunotherapy, targeting with high-specifity and affinity two motifs in the MTBR that form the core sequences required for pathological aggregation of tau. Given the unique binding characteristics, high binding affinity and functional pre-clinical activity of E2814, further clinical investigation is warranted to test whether the antibody can indeed prevent further aggregation of tau and slow transmission of pathology in AD and potentially other tauopathies.

## Supplementary information

**Additional file 1: Table S1.** Affinity and approximate isoform selectivity of anti-tau monoclonal antibodies raised against Peptides 1 and 2. **Figure S1.** Assessment of antibody selectivity for 4R- and 3R-tau isoforms by dot blot and surface plasmon resonance (Biacore). **Figure S2.** E2814 antibody humanisation from mouse 7G6 – schematic representation of the strategy for E2814 generation. **Figure S3.** Substitution scanning of 7G6 antibody affinity requirements on the wild-type tau peptide _1_KDNIKHVPGGGSVQI_15_. **Figure S4.** Selectivity of 7G6 and E2814 antibodies. **Figure S5.** Immunohistochemical labelling by 7G6 of tau fibrillar inclusions in tauopathy brains. **Figure S6.** Immunohistochemical labelling of brain tau by E2814. **Figure S7.** Immunogold labelling of AD filaments with mouse 7G6 antibody. **Figure S8.** E2814 immunogold labelling of tau filaments from different tauopathies. **Figure S9.** Seeding capacity of different tau fragments. **Figure S10.** Western blots from the seed-injection study to assess the efficacy of 7G6 in vivo. **Table S2.** Pharmacokinetic data from the intrahippocampal seed injection study. **Figure S11.** Immunoprecipitation of recombinant aggregated P301S tau. **Supplementary Materials and Methods**.

## Data Availability

All data generated or analysed during this study are included in this published article [and its supplementary information files].

## Abbreviations

3R-tau: Tau isoforms with three microtubule-binding repeat domains
4R-tau: Tau isoforms with four microtubule-binding repeat domains
AD: Alzheimer’s disease
APP: Amyloid precursor protein
BSA: Bovine serum albumin
CB: Coiled body
CBD: Corticobasal degeneration
CDR: Complementarity determining region
ELISA: Enzyme-linked immunoassay
FBS: Foetal bovine serum
FTD: Frontotemporal dementia
i.p.: Intraperitoneal
MAPT: Microtubule-associated protein, tau
MT: Microtubule
MTBR: Microtubule-binding region
NFT: Neurofibrillilary tangle
PiD: Pick’s disease
PIPES: Piperazine-N,N′-bis(2-ethanesulphonic acid)
PSP: Progressive supranuclear palsy
R1,R2,R3,R4: First, second, third and fourth microtubule-binding repeat domain, respectively
RU: Response units
SPR: Surface plasmon resonance
TA: Tufted astrocyte
TRIM21: Tripartite motif protein 21
