# Supplementary material for: Pre-clinical characterisation of E2814, a high-affinity antibody targeting the microtubule-binding repeat domain of tau for passive immunotherapy in Alzheimer’s disease
Source: Acta Neuropathol Commun. 2020 Feb 4;8:13. doi: 10.1186/s40478-020-0884-2 (PMC7001291; doi:10.1186/s40478-020-0884-2)
Supplement: Supplementary file 1 — Additional file 1: Table S1. Affinity and approximate isoform selectivity of anti-tau monoclonal antibodies raised against Peptides 1 and 2. Figure S1. Assessment of antibody selectivity for 4R- and 3R-tau isoforms by dot blot and surface plasmon resonance (Biacore). Figure S2. E2814 antibody humanisation from mouse 7G6 – schematic representation of the strategy for E2814 generation. Figure S3. Substitution scanning of 7G6 antibody affinity requirements on the wild-type tau peptide 1KDNIKHVPGGGSVQI15. Figure S4. Selectivity of 7G6 and E2814 antibodies. Figure S5. Immunohistochemical labelling by 7G6 of tau fibrillar inclusions in tauopathy brains. Figure S6. Immunohistochemical labelling of brain tau by E2814. Figure S7. Immunogold labelling of AD filaments with mouse 7G6 antibody. Figure S8. E2814 immunogold labelling of tau filaments from different tauopathies. Figure S9. Seeding capacity of different tau fragments. Figure S10. Western blots from the seed-injection study to assess the efficacy of 7G6 in vivo. Table S2. Pharmacokinetic data from the intrahippocampal seed injection study. Figure S11. Immunoprecipitation of recombinant aggregated P301S tau. Supplementary Materials and Methods. [file 40478_2020_884_MOESM1_ESM.pdf]

“Pre-clinical characterisation of E2814, a high-affinity antibody targeting the microtubule-binding repeat domain of tau for passive immunotherapy in Alzheimer’s disease”

Malcolm Roberts, PhD; Ioanna Sevastou, PhD; Yoichi Imaizumi, PhD; Kavita Mistry, MSc; Sonia Talma, MSc; Madhurima Dey, MSc; Jane Gartlon, PhD; Hiroshi Ochiai, PhD; Zhi Zhou, PhD; Shigeru Akasofu, PhD; Naoki Tokuhara, PhD; Makoto Ogo, PhD; Muneo Aoyama, MSc; Hirofumi Aoyagi, PhD; Kate Strand, MSc; Ezat Sajedi, PhD; Kishan Lal Agarwala, PhD; Jared Spidel, PhD; Earl Albone, PhD; Kanta Horie, PhD; James Staddon, PhD; Rohan de Silva, DPhil

Supplementary Data

Table S1

Clone	Antigen	Isotype	Wild Type 2N4R tau KD(M)	4R isoform binding	3R isoform binding
**1D10**	Peptide 1	IgG2b, k	1.5×10^{-8}	***	n.b.
**2G9**	Peptide 1	IgG1, k	2.0×10^{-8}	***	n.b.
**3E9**	Peptide 1	IgG2b, k	2.7×10^{-8}	***	n.b.
**4E7**	Peptide 1	IgG2b, k	2.2×10^{-8}	***	n.b.
**6B2**	Peptide 2	IgG1, k	1.3×10^{-9}	***	n.b.
**8E5**	Peptide 2	IgG2b, k	3.9×10^{-10}	***	*
**4E6**	Peptide 2	IgG1, k	1.0×10^{-9}	**	*
**5D1**	Peptide 2	IgG1, k	9.0×10^{-10}	**	*
**1F1-1F6**	Peptide 2	IgG2a, k	4.1×10^{-10}	***	**
**1F1-1G5**	Peptide 2	IgG2a, k	4.7×10^{-10}	***	**
**5H7**	Peptide 2	IgG2a, k	6.1×10^{-10}	***	**
**7G6**	Peptide 2	IgG2b, k	5.2×10^{-11}	***	***

(*** strong binding; ** moderate binding; * weak binding; n.b. no binding)

Table S1: Affinity and approximate isoform selectivity of anti-tau monoclonal antibodies raised against Peptides 1 and 2.

Figure S1

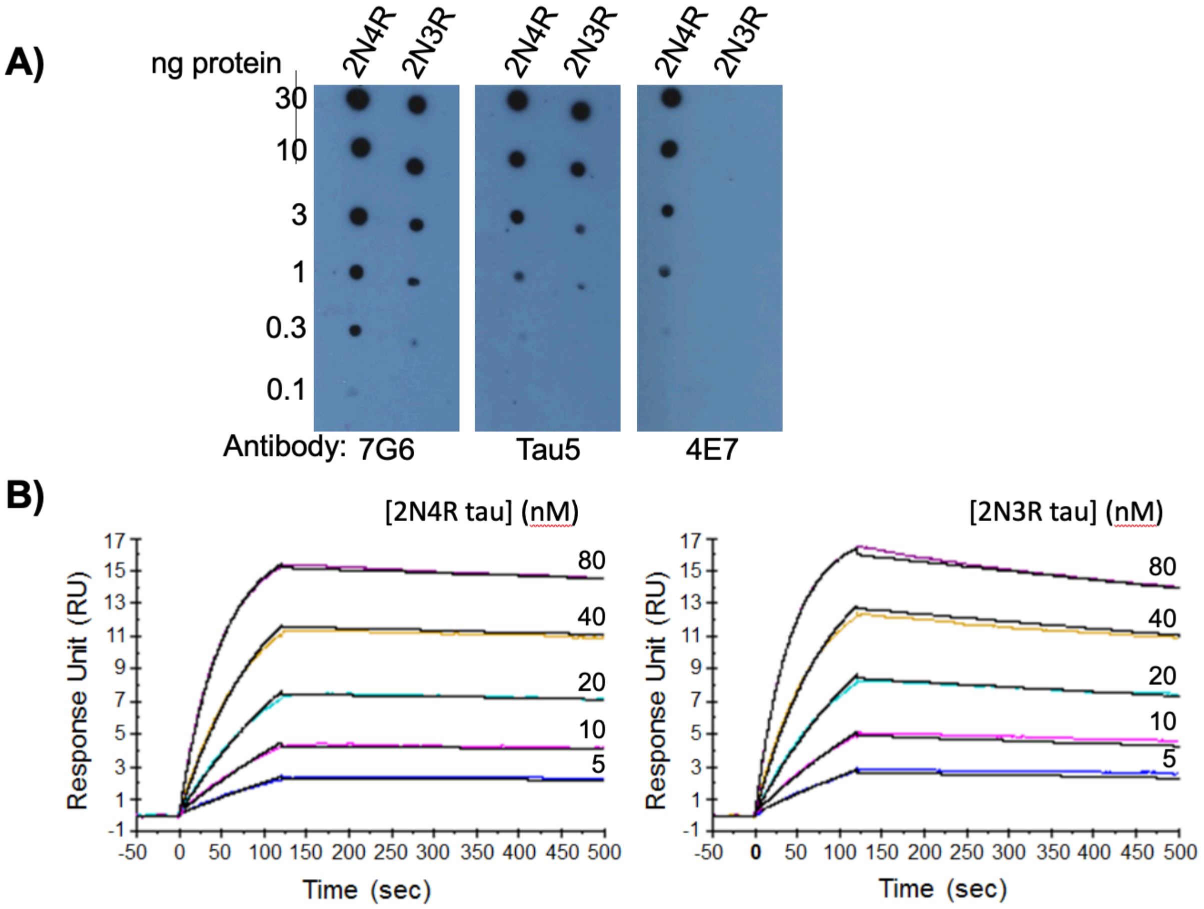

Antigen	k_a(1/Ms)	k_d(1/s)	K_D (M)
2N4R tau	2.46×10^{5}	1.15×10^{-4}	4.66×10^{-10}
2N3R tau	2.86×10^{5}	3.63×10^{-4}	1.27×10^{-9}

Figure S1. Assessment of antibody selectivity for 4R- and 3R-tau isoforms. (A) Decreasing amounts of 2N4R or 2N3R recombinant tau (as indicated) were spotted onto nitrocellulose membranes. Once dry, the membranes were blocked and then probed for tau using 7G6 (left panel), Tau-5 that binds to the mid-domain (middle panel) and 4E7 (right panel) a 4R-tau specific antibody. (B) Quantitative assessment of E2814 affinity to recombinant 2N4R and 2N3R tau was performed using surface plasmon resonance (Biacore). E2814 antibody was immobilised onto the surface of a Protein A/G sensor chip. Different concentrations of recombinant tau protein were passed over the chip to calculate the association rate constant (k_a), dissociation rate constant (k_d) and equilibrium rate constant (K_D).

Figure S2

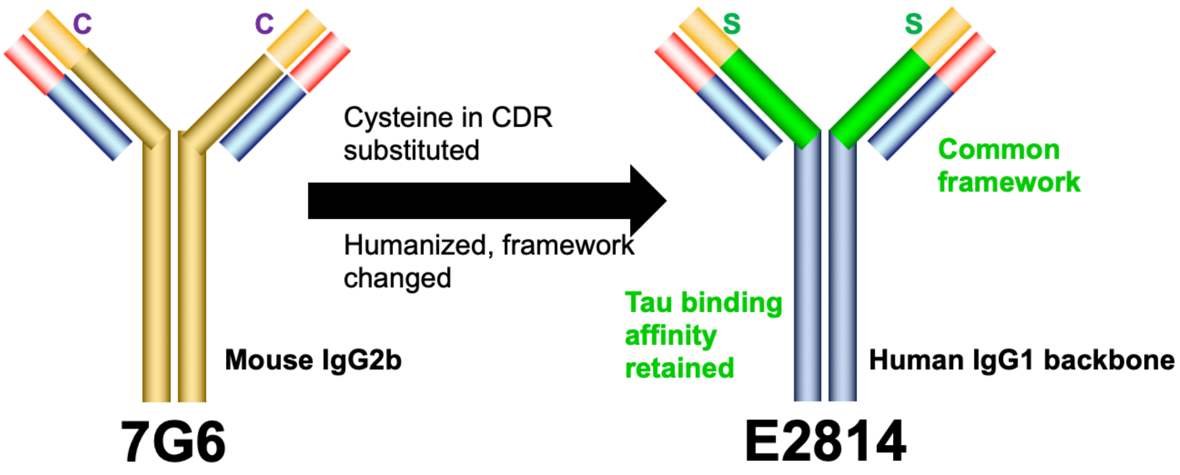

Figure S2. E2814 antibody humanisation from mouse 7G6 – schematic representation of the strategy for E2814 generation. A cysteine residue in complementarity-determining region (CDR) 2 of the murine 7G6 heavy chain was substituted to a serine. All CDR’s were then grafted onto a human IgG_1 backbone with human IGHV3 and IGKV1 frameworks to yield the E2814 antibody. The ability of E2814 to bind tau was comparable with that of 7G6.

Figure S3

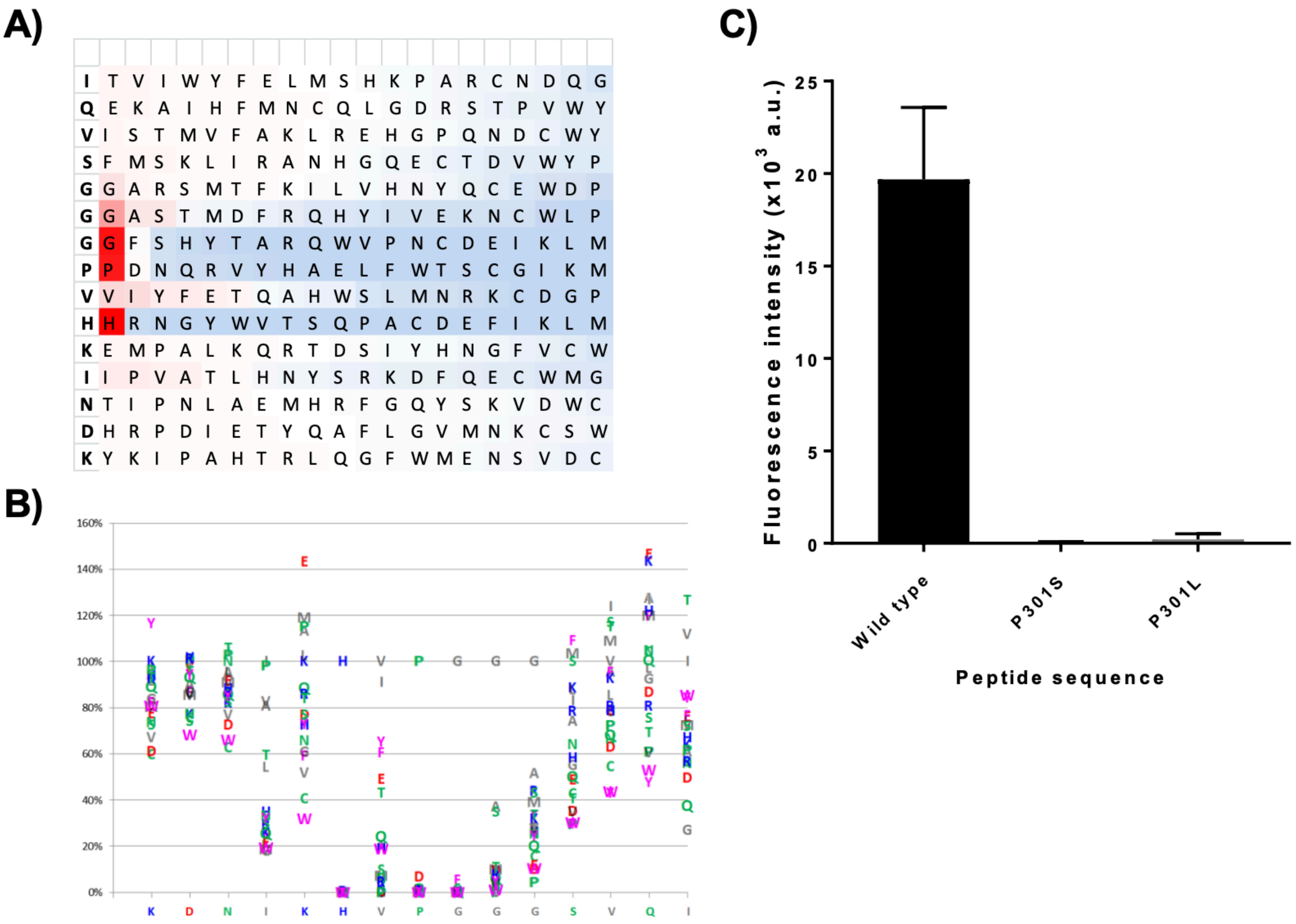

Figure S3. Substitution scanning of 7G6 antibody on the wild-type tau peptide $_1$KDNIKHVPGGGSVQI$_{15}$. The 7G6 antibody has a high requirement for residues H_6, P_8 and G_9 to bind effectively. These residues correspond to H_{299}, P_{301} and G_{302} in the full length 2N4R tau protein. The V_7 residue and amino acids C-terminal to the core HVPG epitope sequence in the peptide also exerted influence on 7G6 binding. (A) Fluorescence intensity values were obtained from the chip image and a heat map of binding was obtained. The preference of binding for given amino acids was colour-coded (red for preferred amino acids; blue, less preferred). (B) A substitution matrix and amino acid plot was generated by dividing the spot intensity of a given peptide by the averaged spot intensities of all twenty peptides at the same position. (C) A plot of fluorescence intensity against P_8 substitutions shows unequivocally how 7G6 binding would be lost in the primary tau sequence containing either the P301S or P301L mutations.

Figure S4

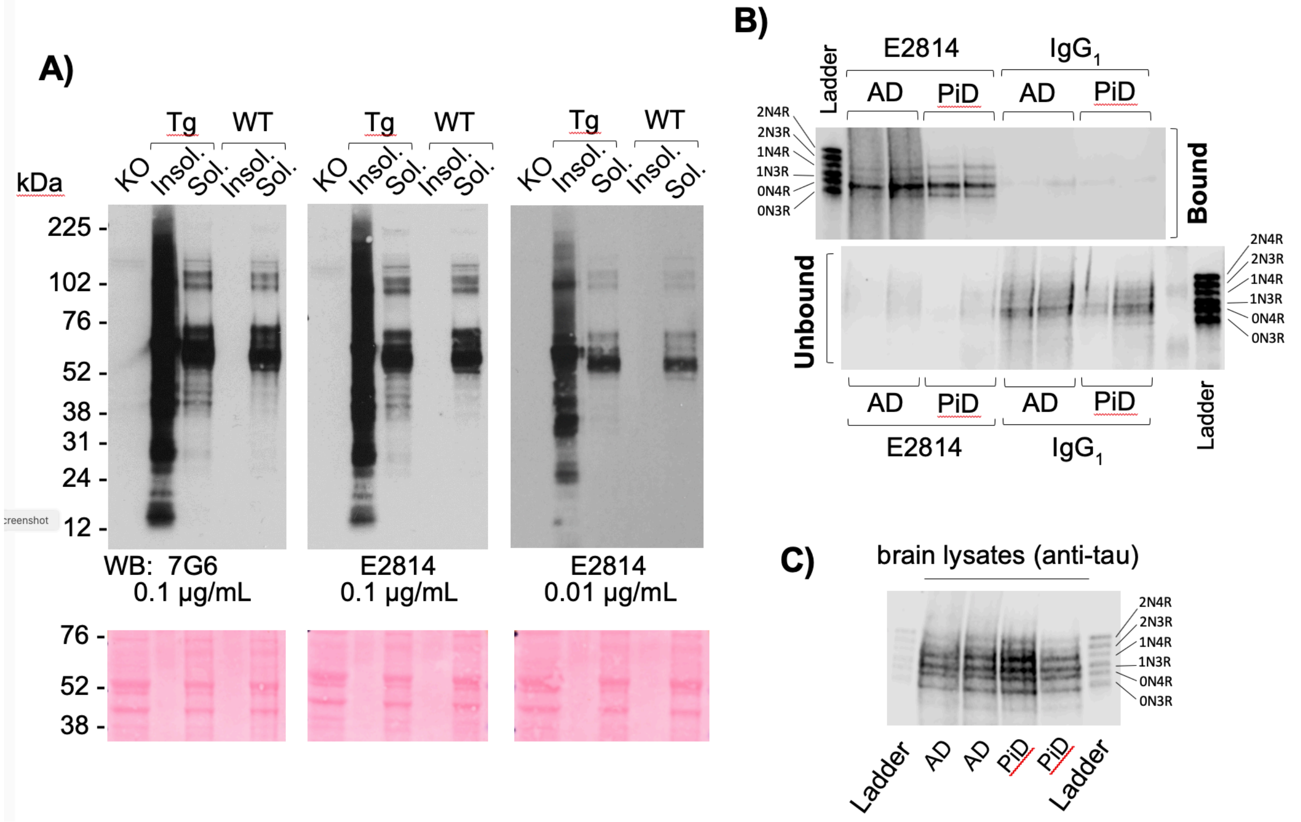

Figure S4. Selectivity of 7G6 and E2814 antibodies. Immunoblotting (upper panels) was performed with 7G6 or E2814 at 1 µg/mL to determine their specificity for tau. (A) Samples from a tau knockout (KO) mouse brain, as well as sarkosyl-insoluble (Insol.) and soluble (Sol.) brain fractions from a rTg4510 (Tg) mouse overexpressing mutant P301L 0N4R tau or wild type (WT) controls were immunoblotted. Fifteen µg of total protein was loaded on the gel for fractions containing soluble tau. For the insoluble tau fractions, pellets were consistently resuspended in SDS-containing sample buffer in one tenth of the volume of starting material (0.2 mL per 8 mg of total protein) and 15 µL was then loaded on the gel. Blots were probed with 7G6 and E2814 antibodies as indicated. No tau was detected in the KO lane whereas a large smear of tau was present in the insoluble fraction of the rTg4510 mouse sample but not wild type. Soluble tau was detected in both Tg and WT mice as expected. A similar pattern is evident for 7G6 and E2814 with additional faint immunoreactivity of higher molecular weight protein observed in the KO sample indicating minor non-tau binding capabilities. Lower panels show Ponceau staining of respective membranes indicating levels of total protein in each lane. (B) Western blotting of Alzheimer's disease (AD; n = 2) and Pick's disease (PiD; n = 2) brain lysates immunoprecipitated with E2814 or IgG_1 isotype control (10 µg antibody/ 50 µg protein of brain lysate). Immunoprecipitates (IP) were dephosphorylated with λ phosphatase and then run on 10% gels together with the immunodepleted flow-throughs. Immunoblotting was performed with anti-tau rabbit polyclonal antibody (K9JA, DAKO). E2814 potently immunoprecipitated both 3R and 4R isoforms of the brain lysates. (C) Immunoblot of whole brain lysates probed with anti-tau rabbit polyclonal antibody (K9JA, DAKO).

Figure S5

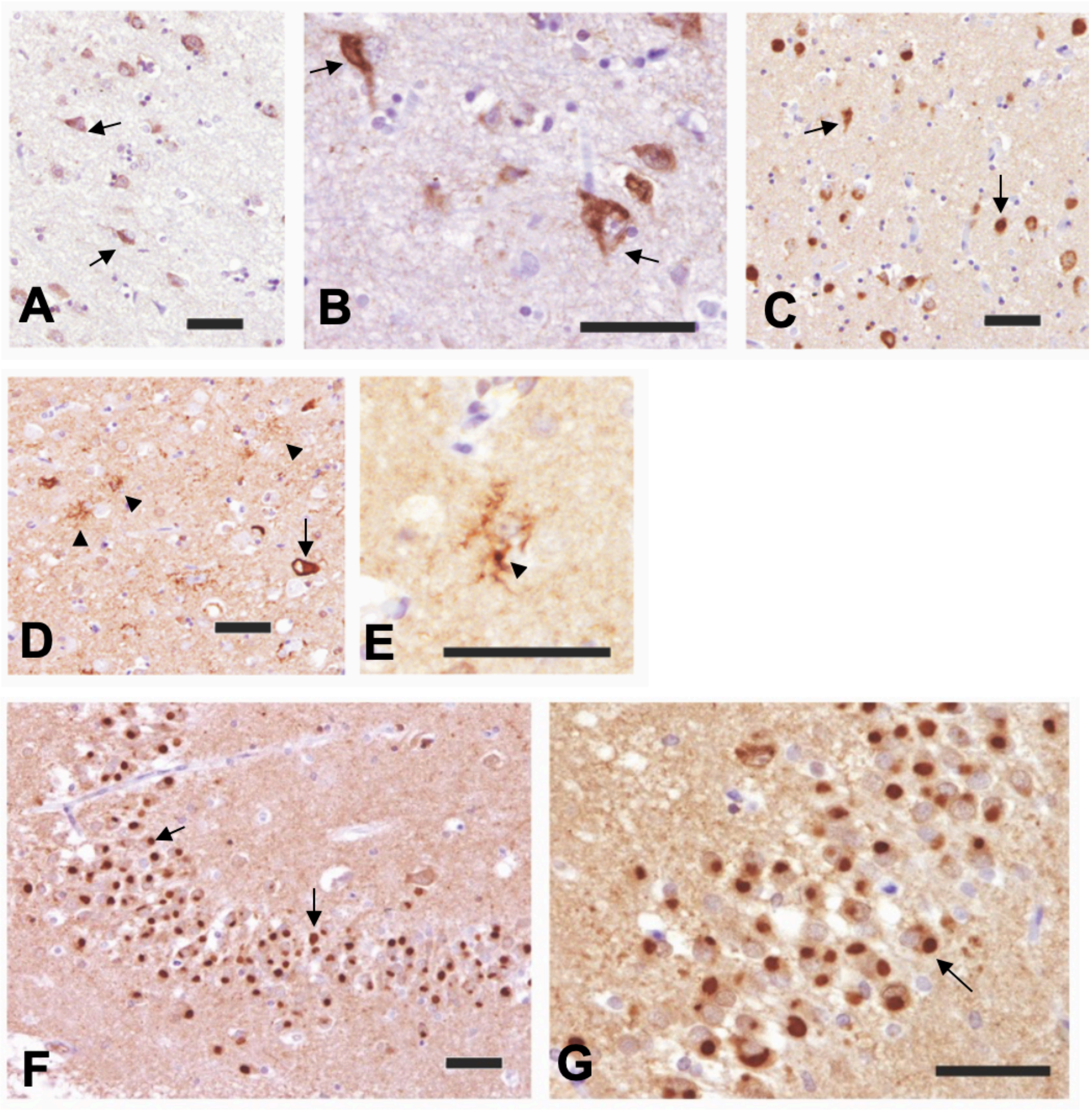

Figure S5. Immunohistochemical labelling by 7G6 of tau fibrillar inclusions in tauopathy brains. Human tissue sections from tauopathy brains were stained with the murine 7G6 antibody at 0.33 µg/mL (A, B): Frontal cortex from AD brain showed robust labelling of neurofibrillary tangles (arrows). (C, D, E): Frontal cortex from a PSP patient demonstrated widespread staining of neurofibrillary tangles (arrows) and tufted astrocytes (arrowheads). (F,G): Hippocampus from a PiD patient exhibited strong staining of Pick bodies (arrows). Scale bars = 50 µM.

Figure S6

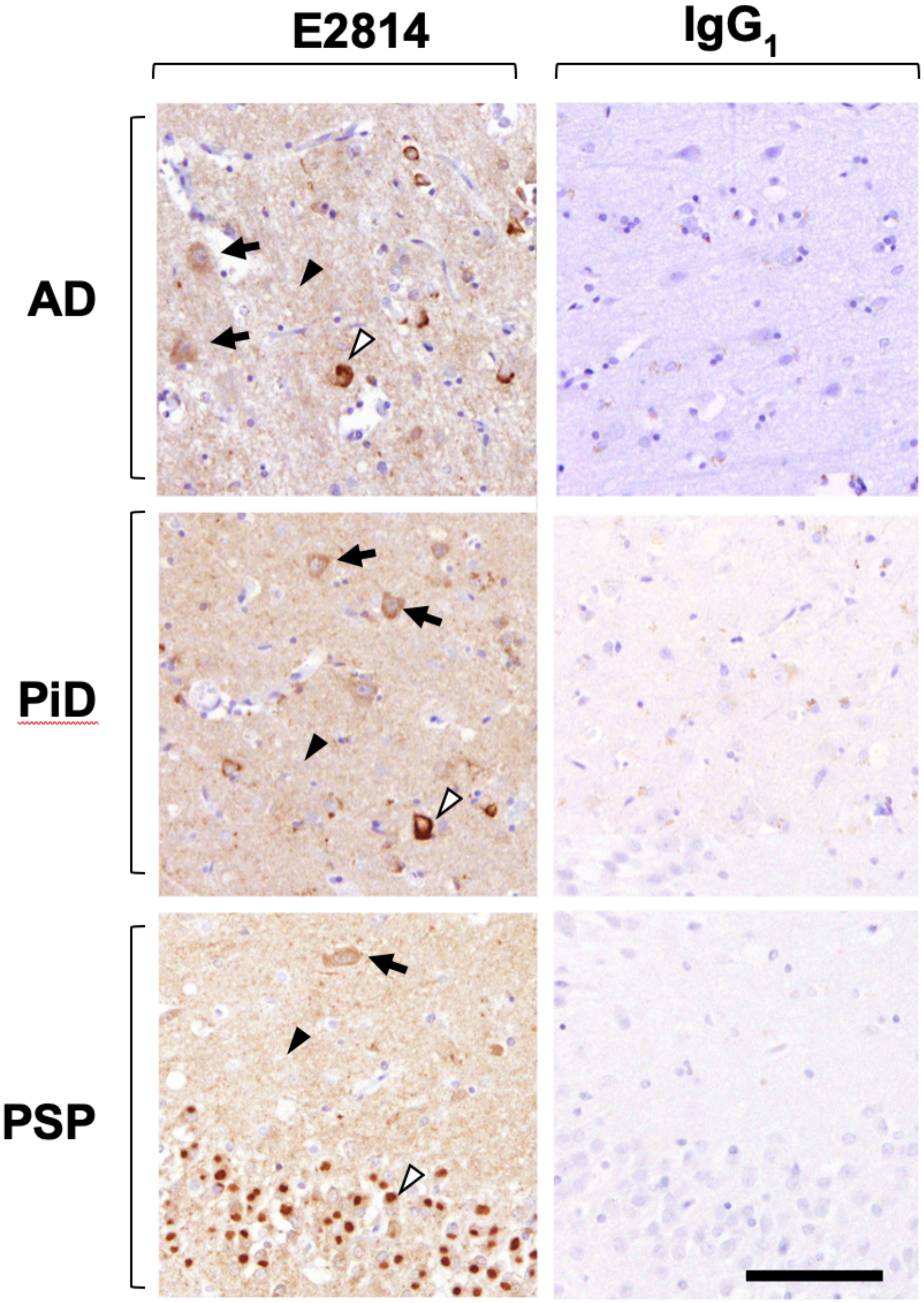

Figure S6 Immunohistochemical labelling of brain tau by E2814. Human tissue sections from tauopathy brains were stained with E2814 antibody at 0.5 µg/mL: Apart from neurofibrillar inclusions (white arrowheads), frontal cortex from AD and PSP and hippocampus of PiD brains showed diffuse E2814 staining of tau in neurons (arrows) and neuopil (black arrowheads) with complete absence of staining with human IgG_1. Brown colouration in IgG_1 control sections is due lipofuscin. Scale bar = 50 µM.

Figure S7

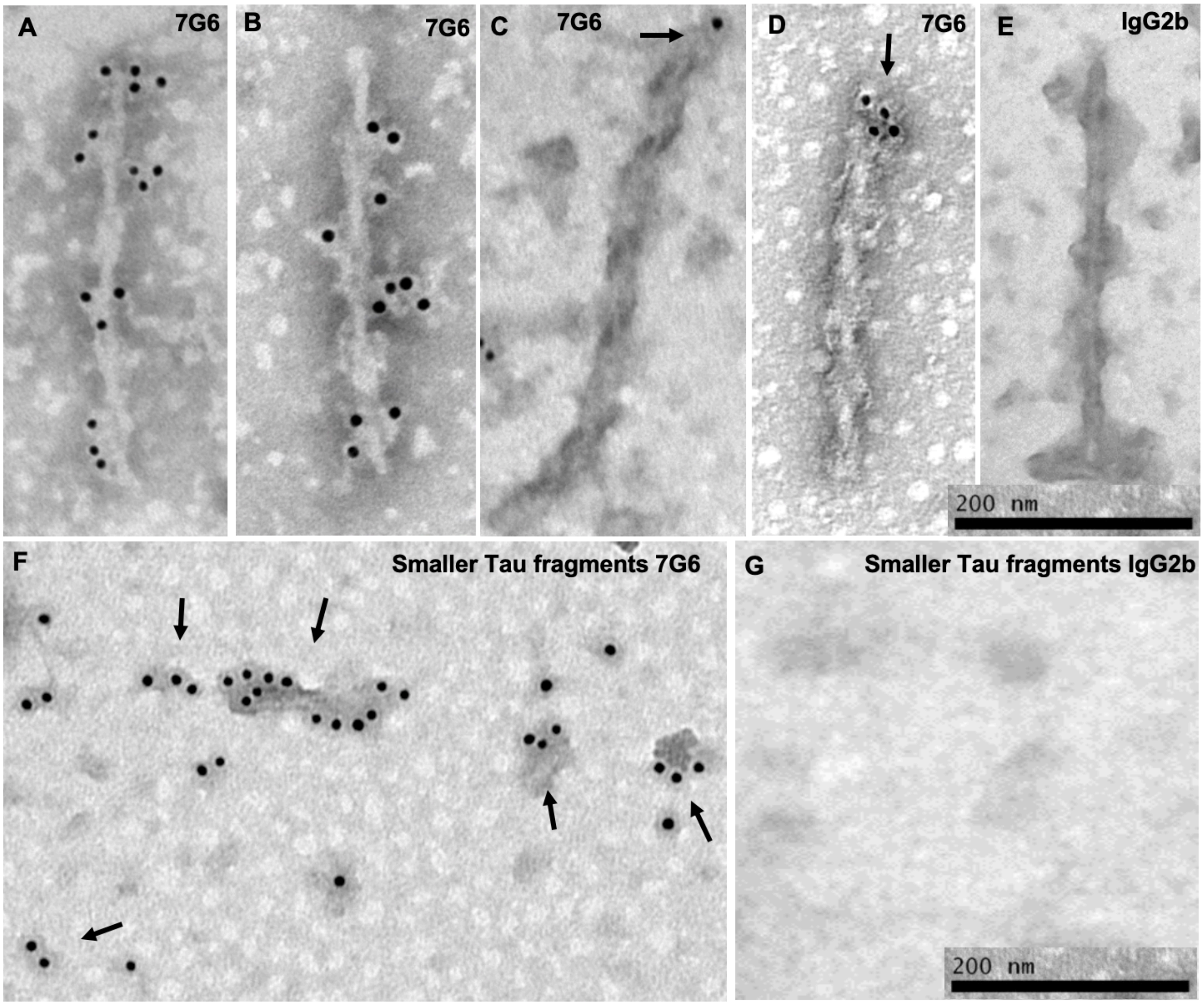

Figure S7. Immunogold labelling of AD filaments with mouse 7G6 antibody. Representative electron microscope images of tau fibrils isolated from AD patient frontal cortex sarkosyl-insoluble fraction. 7G6 or IgG_{2b} isotype control were used at 1 µg/mL. Secondary anti-mouse 10 nm gold conjugated antibody was used at a 1:50 dilution. (A, B) 7G6 could bind the entire length of many tau fibrils but to some paired helical filaments (PHFs) binding (arrows) was limited to the ends (C,D) or completely absent. (F): 7G6 specifically binds to smaller structures on the EM grids that may represent tau fibril fragments or tau oligomers (arrows). No binding was observed for the IgG_{2b} control antibody to filaments (E), or smaller fragments (G). Scale bar = 200 nm.

Figure S8

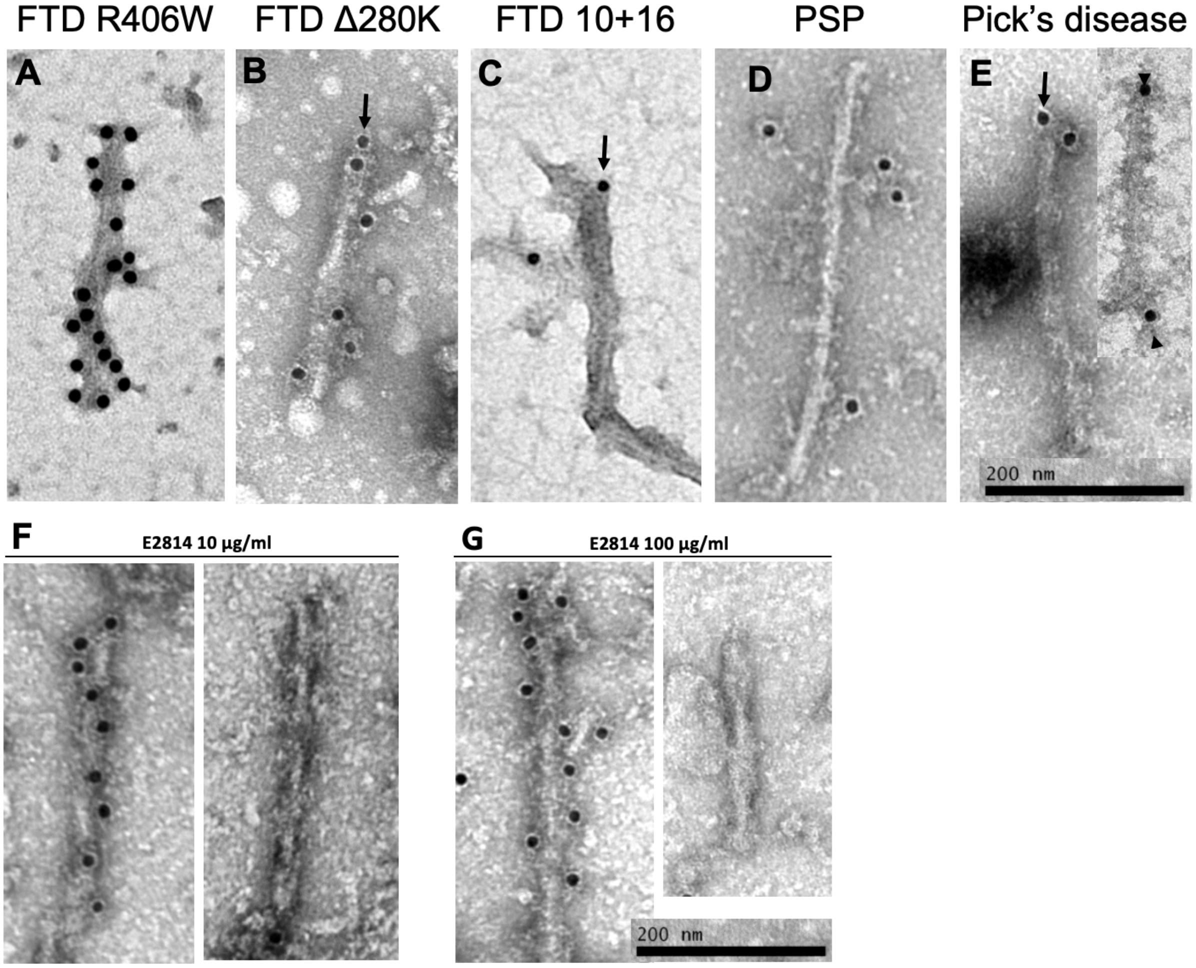

Figure S8. E2814 immunogold labelling of tau filaments from different tauopathies. Representative electron microscope images of tau fibrils isolated from different tauopathy brain samples. E2814 was used at 10 µg/mL (or 100 µg/mL in G) . Secondary anti-human 12 nm gold conjugated antibody was used at a 1:25 dilution. (A) E2814 produces robust decoration of tau fibrils isolated from the frontal cortex of a frontotemporal dementia patient with the *MAPT* missense mutation R406W. (B) E2814 also stains tau fibrils isolated from the frontal cortex of a frontotemporal dementia patient with the MAPT Δ280K deletion. A consistent staining pattern is observed on these fibrils with almost always one end of each fibril being decorated with E2814 (arrow). (C) Only some end-labelling (arrow) was observed of a few tau fibrils isolated from the frontal cortex of a frontotemporal dementia patient with the IVS10 + 16 mutation that leads to increased incorporation of the exon 10. (D) In PSP E2814 binding was not observed on isolated tau fibrils. (E) In fibrils isolated from a Pick's disease patient frontal cortex, binding was not observed through the length of tau fibrils. E2814 did however bind some Pick's tau fibril ends (arrows). (F,G): Representative fibrils from an Alzheimer's disease brain demonstrating how E2814 produces a similar pattern of staining whether used at 10 µg/mL or 100 µg/mL. In (F) two Alzheimer's fibrils stained with the selected concentration of E2814 10 µg/mL are shown. In (G) a very saturated concentration of E2814 is used (100 µg/mL) and the pattern of E2814 staining of Alzheimer's fibrils is similar to (F). No increased, additional staining was observed with 100 µg/mL of antibody. Scale bar = 200 nm.

Figure S9

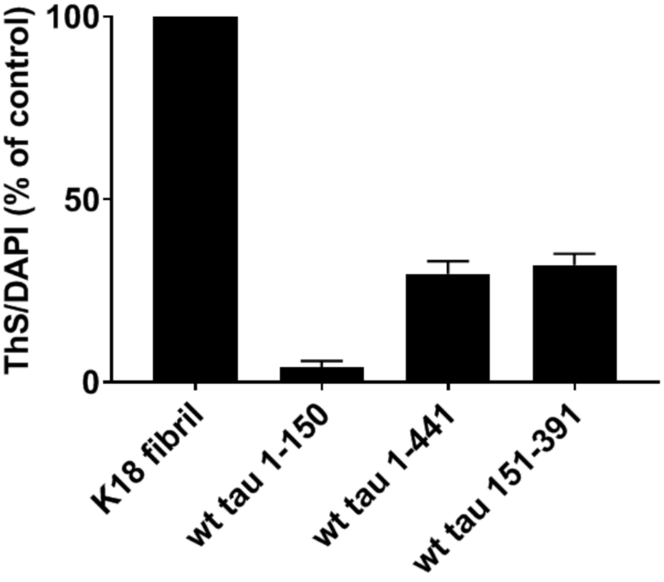

Figure S9. Seeding capacity of different tau fragments. N-terminally truncated tau (wt tau 1-150) monomer showed a reduced seeding activity compared to MTBR-containing K18 fibril, wt tau 1-441 or 151-391 monomers. HEK cells overexpressing 0N4R P301S tau were incubated with the tau seeds as indicated and thioflavin S (ThS) fluorescence was measured. The percentage of ThS/DAPI relative to K18 fibril data is plotted (K18 fibril = 100%, seed (-) = 0%, mean ± SEM, 3 independent experiments).

Figure S10

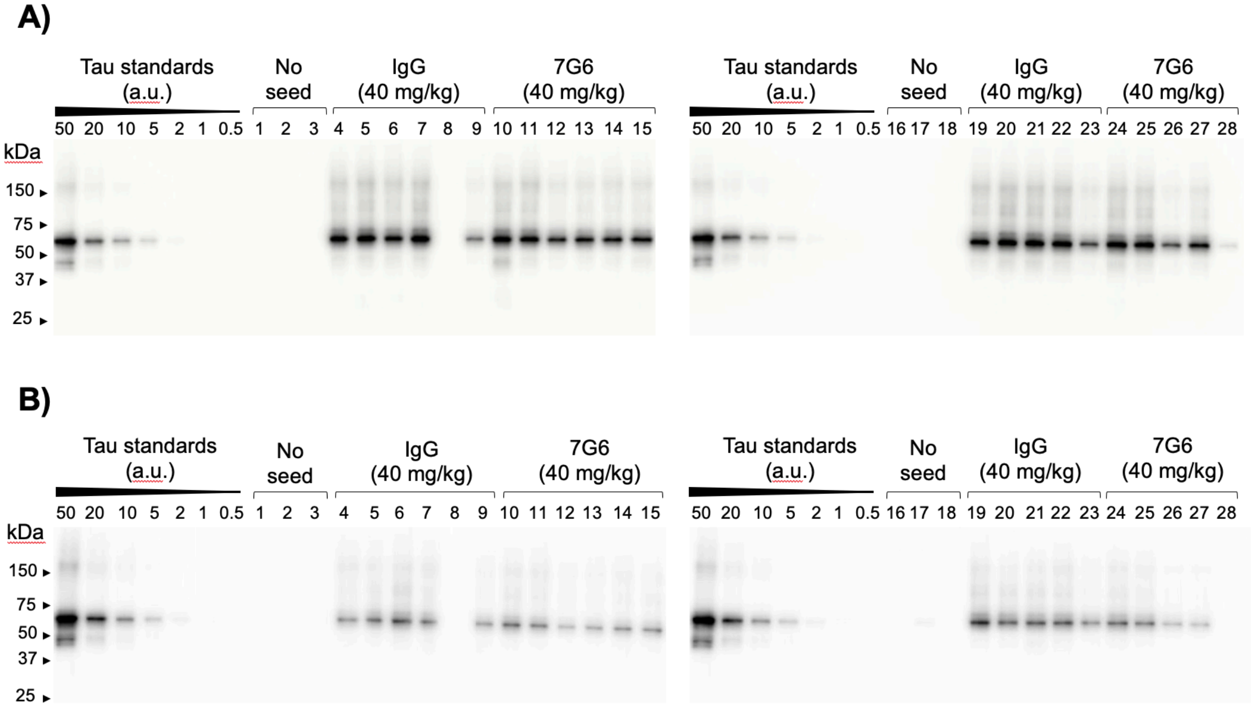

Figure S10. Western blots from the seed-injection study to assess the efficacy of 7G6 in vivo. Sarkosyl-insoluble fractions from the ipsilateral hippocampus (A) or contralateral hippocampus (B) of the seed injection in vivo study were resolved by SDS-PAGE and proteins transferred to nitrocellulose membranes. Tau was then detected by probing membranes with the HT7 antibody. Identical tau standards derived from the insoluble fraction of spinal cord from aged P301S mice were also resolved on each gel to allow quantitation in arbitrary units (a.u.). Animals (as indicated) 1–3 and 16–18, no seed control; 4–9 and 19–23, treated with IgG at 40 mg/kg i.p.; 10–15 and 24–28, treated with 7G6 at 40 mg/kg i.p. Quantification of the blots are presented in Fig. 7.

Table S2

Animal #	CSF 7G6 concentration (ng/mL)	CSF/Plasma ratio (%)	7G6 plasma concentration (µg/mL)
1	124	0.0608	204
2	145	0.0668	217
3	251	0.1157	217
4	151	0.0604	250
5	141	0.0621	227
6	211	0.0818	258
7	142	0.0696	204
8	173	0.0759	228
9	178	0.0748	238
10	161	0.0644	250
11	207	0.0995	208
**Mean**	171.27	0.08	227.36
**SD**	38.04	0.02	19.39

Table S2. Pharmacokinetic data from the intrahippocampal seed injection study.

Figure S11

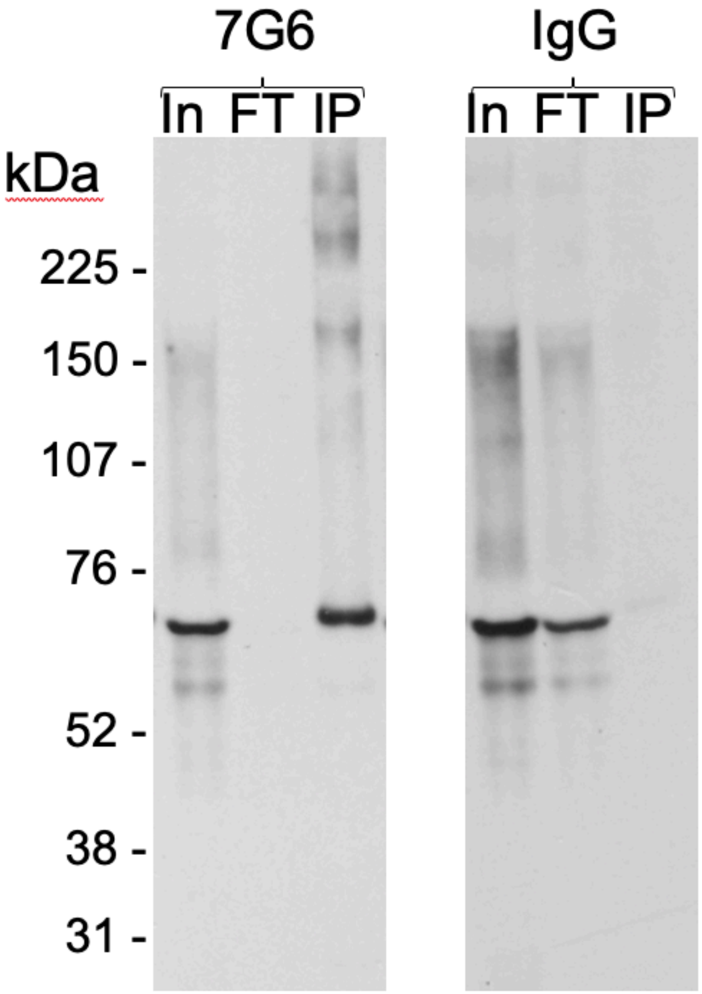

Figure S11. Immunoprecipitation of recombinant aggregated P301S tau. Aggregated recombinant P301S tau seeds described in the in vivo seed injection experiment were used for immunoprecipitation. Approximately 0.8 µg of aggregated tau seeds were incubated with 2 µg of either 7G6 or control IgG antibody as indicated in a total volume of 0.4 mL (Input, In). Bound complexes were captured with Protein A sepharose beads and unbound material (flowthrough, FT) was retained. Protein was eluted from the beads (immunoprecipitate, IP) by boiling in SDS-containing sample buffer. Volume equivalents of denatured samples were then resolved on 4–12% SDS-PAGE gels and proteins were transferred to nitrocellulose membranes. The blot was then probed with a rabbit polyclonal anti-tau antibody (K9JA, Dako) that recognises the C-terminal half of human tau protein.

Supplementary Materials and Methods

Measurement of 7G6 antibody in mouse plasma and CSF

To determine the levels of 7G6 antibody in mouse plasma and CSF, a 96-well multi-array plate (Meso Scale Discovery, L15XA-3) was coated overnight at 4 °C with 50 ng per well of recombinant human 2N4R tau in buffer containing 0.1 M sodium acetate (pH 7) and 0.15 M NaCl. The plate was then washed three times with wash buffer (Tris-buffered saline pH 8, containing 0.05% Tween 20) and then blocked for 1 hour at room temperature in blocking buffer: 0.01 M sodium phosphate pH 7.4, 0.138 M NaCl, 0.0027 M KCl, 0.05% Tween 20, 0.4% Block Ace (Bio Rad), 0.5% BSA, 0.01 M EDTA and 0.05% ProClin 150 (Sigma). Plates were washed a further three times in wash buffer before addition of 50 µL of either plasma (diluted 500-fold in blocking buffer) or CSF (diluted 100-fold in blocking buffer) in duplicate wells and incubated for 2 hours at room temperature. Sample was removed and the plate was washed another five times in wash buffer. Then, a rabbit anti-mouse IgG secondary antibody (Invitrogen, A27022) labelled with Sulfo-tag reagent (Meso Scale Discovery, R91AO-1) was added to each well to bind retained 7G6 antibody. The plate was washed again five times in wash buffer before the addition of 150 µL MSD Read Buffer T (4x) (Meso Scale Discovery, R92TC). The plate was read and data collected using a Sector Imager 6000 instrument (Meso Scale Discovery). To quantitate levels of 7G6 in plasma or CSF, calibration curves were constructed in parallel using known quantities of antibody.

Comparison of E2814 binding to 4R or 3R tau isoforms

For the dot blotting, different amounts of recombinant 2N4R or 2N3R tau (Enzo Life Sciences) were spotted onto a nitrocellulose membrane. Once dry, the blots were blocked and then probed with different primary mouse monoclonal antibodies at 1 µg/mL. The same procedure was followed as the western blotting of fractionated mouse tissue samples described below.

The more quantitative molecular interaction experiments to thoroughly assess E2814 were performed using a Biacore S200 instrument (GE Healthcare). First, recombinant human His-tagged 2N4R and 2N3R tau proteins were prepared using the Musaibo-kun SI cell free expression system (RIKEN) according to manufacturer's instructions. Proteins were then purified by Ni-affinity chromatography followed by reverse-phase HPLC chromatography. Then, two series S CM5 sensor chips (GE Healthcare) in separate flow cells were coated with Protein A/G (Thermo Scientific) using an amine coupling kit (GE Healthcare) which resulted in Protein A/G being immobilised at a high surface density (2360 response units (RU) on each chip).
For antibody capture, E2814 was diluted to a concentration of 0.051 µg/mL in running buffer (10 mM HEPES, 200 mM NaCl and 0.05% Surfactant P20) and passed over the immobilised Protein A/G chip surface at a flow rate of 30 µL/min for 2 minutes at 25 °C. Then, to measure binding kinetics, five different concentrations of recombinant tau protein ranging from 5 to 80 nM were injected over the flow cells. Binding interactions were

monitored in running buffer over a 120-second association period and a 600-second dissociation period at 30 µL/min. Following each cycle, the chip surface was regenerated by injection of 10 mM glycine-HCl, pH2. All binding sensorgrams were collected at 25 °C. Kinetic parameters (k_a, association rate constant; k_d, dissociation rate constant; $K_D = k_d/k_a$, equilibrium dissociation constant), were determined by globally fitting the processed binding curves to a 1:1 Langmuir model.

Immunoprecipitation of tau from brain lysates

Brain tissue from Alzheimer's or Pick's disease patients was lysed in Invitrogen cell extraction buffer (Thermo Fisher Scientific) using a hand-held TissueRuptor (Qiagen) with reusable plastic probes followed by centrifugation at 13,000g for 5 min to remove any debris. The supernatants were collected and the BioRad DC protein assay was performed. For immunoprecipitation, 50 µg of lysate protein were combined with 10 µg antibody or IgG_1 isotype control in IP Lysis Buffer (Pierce) and mixed overnight at 4°C. The next day, 25 µl of protein A/G magnetic beads (Pierce) were washed and combined with the antigen/antibody mixture and incubated for 1 h at room temperature with head-over-end mixing. The immunodepleted flow through was collected to confirm depletion of the epitope with Western blotting. The beads were washed twice with 500 µl TBS-T by gently mixing and once with 500 µl purified water and collected in 100 µl of low pH elution buffer (0.1M glycine, pH 2.0) with incubation and mixing for 10 minutes. Following magnetic separation of the beads the supernatant was collected and neutralised with 15 µl of neutralization buffer (1M Tris pH 7.5) for each 100 µl of elute. Western blotting was performed on the immunoprecipitates and immunodepleted samples for tau detection.

Preparation and western blotting of mouse samples

Brain tissue from adult MAPT-null (KO) or rTg4510 mice was crushed in liquid nitrogen and then homogenised in 11 volumes of buffer containing 50 mM Tris pH 7.5, 10% sucrose, 5 mM EDTA, 800 mM NaCl, 1x HALT protease and phosphatase inhibitors (Thermo Scientific), 1 µM Okadaic acid, 0.1 U/mL alpha-2-macroglobulin and 1 mM PMSF. Homogenates were then centrifuged at 6,000 *g* for 15 minutes at 4 °C. Supernatants were retained and the pellets were homogenized again in another 5 volumes of the same buffer and combined with the previous supernatant to form the 'whole homogenate'. A BCA assay (Thermo Scientific) was performed on the whole homogenates to quantify the protein concentration in each sample which were then adjusted to 4 mg/mL. Normalised samples were then centrifuged at 14,000 *g* for 15 minutes at 4 °C and the supernatant was retained as the 'soluble + insoluble (S1)' fraction. Each sample was adjusted to contain 1% sarkosyl (Sigma) and incubated at room temperature for 1 hour. Then, 2 mL (8 mg of protein) of sarkosyl-treated lysate was ultracentrifuged at 160,000 *g* for 30 minutes at 4 °C. The supernatant was retained as the 'sarkosyl-soluble fraction (S2)'. The pellet was then washed in the same homogenisation buffer and ultracentrifuged again. To obtain the 'sarkosyl-insoluble fraction', pellets were solubilised in 0.2 mL (one tenth of the starting volume) of buffer containing 50 mM Tris pH7.4, 2.3% SDS, 1 mM EDTA, 1x HALT protease and phosphatase inhibitors and 1 mM PMSF. All fractions were snap frozen and stored at -80 °C.

For western blotting, a total of 15 µg protein for the S1 and S2 fractions and 15 µL of the sarkosyl-insoluble fractions were separated on 4-12% SDS-PAGE Bis-Tris Novex gels (Invitrogen) and transferred to nitrocellulose membranes. Blots were then incubated in blocking buffer (5% powdered milk in TBS-T) for 1 hour at room temperature before probing overnight at 4 °C with either 7G6 or E2814 antibodies diluted to 0.1 µg/mL also in blocking buffer. Primary antibodies were removed and blots were washed in TBS-T for 1 hour. Blots were then incubated for 1 hour at room temperature with the relevant HRP-conjugated secondary antibodies: for 7G6, goat anti-mouse IgG (Jackson) diluted 1:5000 in blocking buffer; for E2814, goat anti-human IgG (Thermo Scientific) diluted 1:4000 in blocking buffer. Secondary antibodies were removed and membranes were washed again in TBS-T for another hour. Bound antibodies were detected by addition of electrochemiluminescence reagent (GE healthcare) followed by exposure to x-ray film (Fuji).

Epitope substitution scanning

To determine 7G6 antibody sensitivity to single amino acid substitutions within and proximal to the HVPGG binding sequence, a full epitope substitution scanning experiment was performed by PEPperPRINT GmbH (Heidelberg, Germany). One amino acid at a time, each amino acid of the naturally occurring 2N4R tau 15-mer peptide sequence KDNIKHVPGGGSVQI was replaced by all of the other nineteen, naturally-occurring amino acids and synthesised. Substituted peptides along with fifteen unsubstituted peptides were then laser printed in triplicate onto a glass chip. The 7G6 antibody was incubated on the chip at a concentration of 1 µg/mL diluted in PBS containing 10% Rockland blocking buffer (Rockland, MB-070) overnight at 4 °C. Antibody was removed and the chip was washed in PBS containing 0.05% Tween. Next, the chip was incubated with goat anti-mouse IgG DyLight™ 680 (Thermo Fisher, diluted 1:5000) secondary antibody for 45 minutes at room temperature. The chip was washed again as before and fluorescence images were acquired on a LI-COR Odyssey™ imaging system. Microarray data were then analyzed using PepSlide™ Analyser software.
